# Observation of Weibull, Lognormal, and Gamma Distributions in Electrodeposited Cu and Cu-Ag Particles

**DOI:** 10.3390/ma16196452

**Published:** 2023-09-28

**Authors:** Yunkai Sun, Giovanni Zangari

**Affiliations:** Department of Materials Science and Engineering, University of Virginia, Charlottesville, VA 22904, USA

**Keywords:** electrodeposition, nucleation and growth, particle statistics, Lognormal distribution, Weibull distribution, Gamma distribution

## Abstract

In this work, the nearest-neighbor distances and Voronoi cell features of Cu-Ag deposits were analyzed and fitted with Lognormal, Weibull, and Gamma distributions. The nearest-neighbor distance distributions of the samples were compared with those of complete spatially random points, showing spatial inhomogeneity due to the nucleation exclusion effect. The radial distribution function was calculated, showing both influences from the grain size and the nucleation exclusion effect. Voronoi cells were generated based on the shape of the grains. The size, occupancy, and coordination of the Voronoi cells were examined and fitted. The results show that although the Cu-Ag deposits seemed to be governed by the instantaneous nucleation mode, the spatial distribution of the nuclei was more impacted by the nucleation exclusion effect than the Cu-only samples. This behavior is also justified by the grain size distribution generated with Voronoi cell size and occupancy distributions.

## 1. Introduction

Traditionally, the nucleation and growth behavior in electrodeposition systems were investigated by comparing observed potentiostatic transients with the classical model (1968) and the Scharifker–Hills model (S-H model, 1983) [1,2]. Despite the quite simple and straightforward derivations of these two models, which are widespread and successful in explaining the potentiostatic transients at the initial stage of electrodeposition, the microstructural evidence of the fundamental assumptions of the S-H model is not sufficient [3]. Furthermore, a recent observation showed that some systems do not follow the physical picture in the S-H model. For example, Ustarroz et al. reported that Pt particles were mainly grown through the clustering of newly formed Pt nuclei (called secondary nucleation [4]) during electrodeposition of Pt at carbon-coated TEM grids. These potentiostatic transients imply significant deviations from the physics of the S-H model [5]. Although the distributions of features in electrodeposited grains have been observed, the relationship between the nucleation modes from the S-H model and the grain statistics is rarely investigated from a morphological perspective (despite being frequently referred to in explaining the potentiostatic transients [3]).

To understand the nucleation and growth behavior in electrodeposition beyond the potentiostatic transient method, we believe that analyzing the statistical features of the grains at the initial stage of electrodeposition could offer useful insights. The main complexity of electrodeposition nucleation and growth is the nucleation exclusion behavior. The diffusion fields around the particles formed at an early time will reduce the nucleation rate around those particles; such behavior is usually referred to as the nucleation exclusion effect. The area that represents a very low nucleation rate around a single grain is its nucleation exclusion zone. The morphology of the spatial features in electrodeposits should reflect the role of the nucleation exclusion effect, especially in the early stage of electrodeposition.

However, so far, the only systematically examined statistical features of electrodeposited grains are the particle sizes and the nearest-neighbor distances [6,7]. In the literature (summarized below), the morphology of electrodeposits is usually discussed qualitatively, which makes the comparison between different electrodeposition conditions and systems very difficult.

The impact of nucleation exclusion zones on the randomness of the spatial distribution of nuclei has been observed in various works to be reflected by the distribution of the nearest-neighbor (NN) distances and the radial distribution function (RDF) of the nuclei [8,9,10,11,12]. Experimental results show that the degree of inhomogeneity could be influenced by the deposition overpotentials [9] and the supporting electrolyte [13]; increasing the overpotential reduces the NN distances and undermines the randomness of the nuclei, while the addition of a supporting electrolyte will improve the spatial randomness of the nuclei. The impact of nucleation exclusion zones in different systems can be different. For instance, the exclusion effect can extend to the 15th nearest neighbor of Pb particles at vitreous carbon substrates [9], but only to the 2nd NN of Ag grains at boron-doped diamond electrodes [14]. The nucleation events in electrodeposition can last from seconds to tens of seconds, based on direct observation of nuclei with optical microscopes [13,14,15].

Recently, Moehl et al. (2020) observed the in-plane periodicity of Au particles electrodeposited at a Si substrate using grazing incidence small-angle X-ray scattering (GISAXS) with a synchrotron X-ray [16]. To separate the effect of the nucleation kinetics and growth kinetics on the final deposit, a double-pulse deposition was used [13]. A small XRD peak, corresponding to a periodicity (correlation distance) slightly larger than the nearest-neighbor distances of the grains, was observed in the horizontal diffraction profile (along the substrate). Moehl et al. considered the correlation distance to correspond to the periodicity of larger particles, instead of all particles [16].

Besides these experiments, various efforts have been made to mimic such inhomogeneity via simulation. In 1992, Scharifker et al. proposed a method of simulating the nucleation and growth process with progressive and nucleation modes of the S-H model, in which the instantaneous nucleation mode leads to completely spatially random grains while the nucleation exclusion zone during progressive nucleation significantly impacted the spatial homogeneity of the grains [17]. With an experimentally measured nucleation rate, $A_{\eta }N^{f,\infty }$, from the potentiostatic transients [9], Mostany et al. simulated the spatial distribution of grains (RDF and NN distances) from the nucleation process under the influence of nucleation exclusion zones (with the radius ${R_{i}\sim \left(t-\tau_{i}\right)}^{1/2}$), showing good agreement between observed and simulated results (1st–15th NN neighbors) [8,9]. In addition to Scharifker’s approach with hard-cored nucleation exclusion zones, Milchev et al. [6], Kruijt et al. [13], Hyde et al. [14], and Tsakova and Milchev [18] simulated the impact of diffusion fields at a given site with diffused hemispherical diffusion fields from its first nearest nucleus [6,13,14,18] or n-th (n < 5) nearest nuclei [13], showing good agreement with observed values. Simulations by Tsakova and Milchev showed that inhomogeneity increases with nucleation site density [18]. These works, however, did not connect their simulated results with an analytical solution of their model, nor compared their findings with the distributions for spatial features in metallurgy.

To the best of our knowledge, a qualitative understanding of the relationship between deposition conditions and the spatial distribution of the deposit is still lacking. The hard-disk model developed by Torquato et al. offers an analytical solution to the nearest-neighbor distance distribution in a system consisting of impenetrable disks with the same diameter, which cannot describe the distributions in electrodeposition systems [19]. Recently, rooted in the Kolmogorov–Johnson–Mehl–Avrami (KJMA) approach, Tomellini et al. proposed a theory for estimating the RDF and NN distance distribution of nuclei generated at a constant nucleation rate (progressive nucleation) under the influence of nucleation exclusion zones (following a generalized growth law of $R\left(t\right)={\left(\gamma t\right)}^{n/2}$) [20]. This model suggests that the first NN distances could be fitted using a generalized Gamma distribution. However, from our perspective, how to use this model to explain the statistics of the spatial features in actual deposits is still very obscure. Developing an analytical theory for a nucleation and growth model in electrodeposition beyond the S-H model is still an ongoing investigation [5,7,21], with Politi and Tomellini categorizing the strategies of these theories in two fashions: “3D nucleation and growth” and “planar diffusion analogy” [7].

The distribution functions (for example, Lognormal, Weibull, and Gamma distributions) reflect the underlying mechanism of nucleation and growth during material processing, which has been extensively utilized in the field of metallurgy [22]. However, the connection between the distributions and the observed microstructure is still unclear. Regardless, for electrodeposition, we believe that the introduction of these distribution functions could quantify the features observed in electrodeposited particles, which could facilitate future theoretical research in electrochemical nucleation and growth with more statistically significant data. Furthermore, a more quantitative description of the spatial distribution of electrodeposits could facilitate the adoption of emerging data science techniques in the design of electrodeposition processes.

Thus, in this work, we attempt to compare the observed spatial features in electrodeposited Cu or Cu-Ag particles with the Lognormal, Weibull, and Gamma distributions, in the hope that a theory about the spatial distributions in electrodeposited particles could be proposed in the future, with data collected from Atomic Force Microscopy (AFM) [23], Scanning Electron Microscopy (STM) [24,25], and emerging new characterization tools for electrochemists such as Scanning Electrochemical Cell Microscopy (SECCM) [26] and Liquid Cell Transmission Electron Microscopy (TEM) [27].

## 2. Experiments

The Scanning Electron Microscopy (SEM) images used in this paper are from the dataset of our previous works [28,29,30]. A heavily As-doped H-terminated n-Si(001) wafer with a resistivity < 0.005 Ω⋅cm (Silicon Quest International, San Jose, CA, USA) was cut and then cleaned with methylene chloride, acetone, and ethanol. The n-Si(001) pieces were then etched in 30% HF solution and soldered with In-Ga (at room temperature) at metal supports. The depositions were conducted in a typical 3-electrode cell, with a 300 mL beaker as the container, Pt mesh as the anode, n-Si pieces of ~1 cm^2^ as the substrate, saturated mercurous sulfate (SMSE, 0.64 vs. SHE) as the reference electrode, and EG&G PAR 273 potentiostat/galvanostat (ArtisanTG, Champaign, IL, USA) as the potentiostatic control. The deposition bath is associated with components of 10 mM CuSO_4_ + 0.5 M H_2_SO_4_ + 0, 0.1, or 1 mM AgNO_3_. No stirring was permitted during the deposition, and the n-Si(001) substrates were exposed to surrounding illuminations during deposition. The cutoff deposition time for all samples was 5 s. The SEM images were collected with a JEOL JSM-6700F field-emission scanning electron microscope (FE-SEM) (JEOL, Tokyo, Japan).

**Figure 1:**
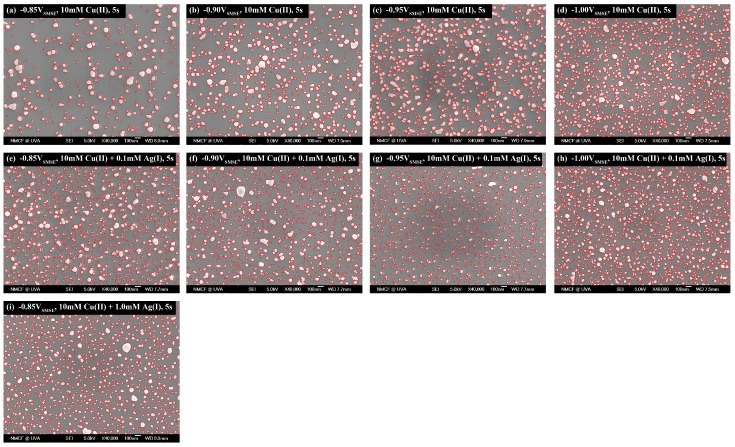
SEM images of the Cu-only and Cu-Ag samples used in this work. Deposition stopped at 5 s for all samples. The red lines indicate the boundary of the grains identified after image processing. The supporting electrolyte was 0.5 M H_2_SO_4_. All the other deposition parameters are listed in the figures. Original SEM images are included in Section S1. Different rows represent different Ag concentrations.

The spatial features in the SEM images (Figure 1) were processed using ImageJ 1.52a. The Voronoi cells were generated based on the shapes of the grains, instead of the weight center of the grains. On the other hand, the n-th rank NN distances were generated based on the weight center of the grains. To avoid interference from the edges of the imaging area, a cutoff distance with the maximum observed distance of a given rank of NN distances was used: only the grains outside this cutoff distance were used for the NN distance distribution. The radial distribution function (RDF) was calculated using a fixed 500 nm cutoff distance since the range of interest was about 500 nm.

## 3. Fitting Functions

### 3.1. Generalized Gamma Distribution

The spatial characteristics of a grain structure often reflect the nucleation and growth behavior of the system [22]. Distributions derived from the generalized Gamma distribution, such as Gamma, Lognormal, and Weibull distributions, are frequently used for the statistics of the features of a grain structure [22,31].

The generalized Gamma distribution is the analytical result for the distributions of the η-th nearest-neighbor distance for a set of completely randomly located points. With $\rho =N^{tot}/V^{tot}$ as the spatial density of the points, the probability of having an η-th nearest neighbor with the length R in an n-dimensional space is a generalized Gamma function of η, n, and ${\left(a\rho \right)}^{1/n}$ [6,32]:

(1) $$p\left(\eta ,R\right)=\frac{{n\left(a\rho \right)}^{\eta }R^{\eta n-1}}{\Gamma \left(\eta \right)}\exp\left(-a\rho R^{n}\right)$$

Note that for the 2D scenario, a=π. The mean and standard deviation of this distribution are $\mu =\frac{1}{(a{\rho )}^{1/n}\Gamma \left(\eta \right)}\cdot \Gamma \left(\eta +\frac{1}{n}\right)$ and $\sigma =\frac{1}{(a{\rho )}^{1/n}}{\left(\frac{\Gamma \left(\eta +\frac{2}{n}\right)}{\Gamma \left(\eta \right)}-{\left(\frac{\Gamma \left(\eta +\frac{1}{n}\right)}{\Gamma \left(\eta \right)}\right)}^{2}\right)}^{1/2}$.

When the nucleation exclusion zone is significant, the spatial distribution of the center of grains will no longer be homogeneous [17,33]. To the best of our knowledge, Tomellini’s model (2017) is the only analytical model for the spatial distribution of electrodeposited particles.

For electrodeposition, Tomellini’s model assumes that (1) the nucleation rate is a constant (progressive mode) and (2) the radius of the nucleation exclusion zone $R\propto \tau^{1/2}$. With these physical setups, Tomellini found that the 1st NN distance distribution, after full coalescence of the nucleation exclusion zones, is a generalized Gamma distribution with n≈1.8 and η≈2.83. However, Tomellini’s model only introduced two of the three fitting parameters; this model did not discuss how to calculate the mean 1st NN distance based on a certain definition of the nucleation exclusion zone with parameters from the mass transport and nucleation kinetics of actual electrodeposition.

In addition to the NN distance distribution, the generalized Gamma distribution is closely related to the particle size distribution of a system. The 2-parameter Weibull distribution is achieved from the generalized Gamma function with η=1, and the Gamma distribution is achieved with n=1. Meanwhile, the Lognormal distribution was proven to be a special case of generalized Gamma distribution when η→∞ [34,35,36]. Therefore, the generalized Gamma distribution could be used to choose the distribution functions for a given set of particle sizes. However, how the generalized Gamma distribution is connected to the physical model of the evolution of particles is still unclear to us [37]. Arbitrarily fitting the data with the generalized Gamma distribution is difficult [34,35,36]. Thus, the generalized Gamma distribution is difficult to use to analyze particle size distribution.

### 3.2. Weibull Distribution—The Size of Fragments with Randomly Partitioned Volumes

According to Rinne [38], several physical models are derived from the Weibull distribution: (1) the weakest link model, wherein the failure probability of the entire system is due to the failure of the weakest (or shortest lifetime) part of the entire system [38]; (2) the degradation process, if the first passage time of the failure for an individual part follows inverse Gaussian distribution; (3) the hazard rate, regarding the wearing of a device; and (4) the “generalized broken stick model”, wherein the size distribution of broken pieces of a finite length stick with an expected size [31,39]. With the fitting parameters $\left(\nu ,k\right)$, the expression of the Weibull distribution of $V_{R}$ is

(2) $$p\left(V_{R}\right)=\frac{k{V_{R}}^{k-1}}{\nu }\exp\left(-\frac{{V_{R}}^{k}}{\nu }\right)$$

Note that the fit from the Weibull distribution is independent of how the particle shape is defined. Assuming a particle with the volume $V_{R}=aR^{n}$, both n and a can be incorporated into the fitting parameters without changing the shape of the fitted curve.

Lourat (1974) found that the radius of particles R fulfilled a Weibull distribution with k=2 [40] (which is also called “Rayleigh distribution” [22]). Fayad et al. simulated grain evolution during an annealing process in a 2D system, leading to a Weibull distribution of grain radius R with k≈2.5 at the steady state [41].

In an analogy to the generalized broken stick model [42], the Weibull distribution could be related to the allocation of depositing species to each nucleus. When the allocation of resources is completely random, with the volume of each particle, $V_{R}$, as the variable of the distribution, the fitting parameters are as follows: k=1 and $v=V^{tot}/N^{tot}$. On the other hand, when the particle radius is completely random, the particle size could be described by the Weibull distribution, with k=3 for $V_{R}\sim R^{3}$ and k=2 for $V_{R}\sim R^{2}$. Figure S1 in Section S2 demonstrates the said process. However, the physical picture of the Weibull distribution in the nucleation and growth problem is still unclear to us, and there could be other explanations for the occurrence of Weibull distribution.

### 3.3. Lognormal Distribution—Law of Proportionate

The Lognormal distribution [43,44,45,46,47,48] is frequently used in generalized nucleation-growth statistics (e.g., bacteria population [49]; shape and size of metallurgical grains [50], colloids [51], and cumulus clouds [52]; pore sizes in soils [53]; commercial firm sizes [54]; or clumping of galaxies [55]), as well as for other aspects, such as survival time [56], paper citations [57], surgical procedure duration [58], and user post length in Internet discussions [59].

Conceptually, the Lognormal distribution will emerge when the values of original random events evolve with respect to steps via multiplication with a series of random parameters. With the fitting parameters $\left(\mu ,\sigma \right)$, the expression of the Lognormal distribution of particle size $V_{R}$ is

(3) $$p\left(V_{R}\right)=\frac{1}{V_{R}\beta \sqrt{2\pi }}\exp\left(-{\frac{1}{2}\left(\frac{\ln\left(V_{R}\right)-\mu }{\sigma }\right)}^{2}\right)$$

Note that the fitting of Lognormal distribution is independent of how the particle shape is defined. If R fulfills the Lognormal distribution of variables $\left(\mu ,\sigma \right)$, then $aR^{n}$ fulfills the Lognormal distribution of $\left(n\mu +\ln a,\;n^{2}\sigma^{2}\right)$.

According to a book by Aitchison and Brown published in 1966 [48], the Lognormal distribution for the grain size of ground material was first proposed by Kolmogoroff (1941), who assumed the partition of each particle is independent of its size at discrete breakage steps and the step number [60,61]. A continuous version of this model associated with a breakage frequency was recently (2003) proposed by Gorokhovski and Saveliev regarding the breakage of liquid droplets (initially of size $R_{0}$) produced by an air blast [62]. In 2008, Bergmann and Bill showed that when the untransformed fraction of the parent phase is proportional to $\exp\left(-{\left(\frac{t}{t_{cI}}\right)}^{2}\right)$, with a constant nucleation rate $I_{0}$ and growth rate $v_{0}$, the grain sizes at the end of the transformation will be similar to the Lognormal distribution [63,64]. Despite the assumption of the observed nucleation rate $I\propto I_{0}\exp\left(-t^{2}\right)$ in their work, which agrees with the apparent nucleation rate for the progressive nucleation mode of the S-H model [65], it is uncertain whether the initial electrodeposits could be expressed with this model (which was derived for grain statistics in complete crystallization).

In terms of electrodeposition, the Lognormal distribution could be achieved for grains that are initially the same size when (1) the average growth and shrink rate of each grain are proportional to its size ($V_{R,(t+dt)}=T\cdot V_{R,(t)}$) but independent of deposition time, and (2) the randomness of the growth and shrink is independent of the size of the grains and deposition time [66,67]. A hypothetical scenario is given in Figure S2 in Section S2. However, the relationship between the Lognormal distribution and the electrodeposition problem is still unknown, and there could be other explanations for the occurrence of Lognormal distribution.

### 3.4. Gamma Distribution—The Size of Voronoi Cells with Completely Randomly Located Points

The Gamma distribution is known to be closely related to the statistics of events fulfilling the Poisson distribution [68]. With the fitting parameters $\left(\alpha ,\gamma \right)$, the expression of the Gamma distribution is

(4) $$p\left(V_{R}\right)=\frac{\gamma^{\alpha }V_{R}^{\alpha -1}}{\Gamma \left(\alpha \right)}\exp\left(-\gamma V_{R}\right)$$

where $V_{R}$ is the volume of the particles at a given dimension (e.g., $V_{R}=\pi R^{2}$ for 2D circular plates). Note that the fitting results will be different if the particle shape is defined differently.

The size distribution of the Voronoi cells ($V_{R}$) associated with completely spatially random points (generated from a homogeneous Poisson point process), called Poisson–Voronoi tessellation, could be described by the Gamma distribution [69,70]. In this case, the fitting parameter could be separated into $\gamma =\rho \gamma^{\prime }$, with $\rho =1/\bar{V}=N^{tot}/V^{tot}$ as the density of the random points. While the 1D scenario of the tessellation analytically fulfills a Gamma distribution, the scenarios at higher dimensions are not analytically available but empirically successful: α=γ=2 for a 1D system, α≈γ≈3.5 for a 2D system, and α≈γ≈5 for a 3D system [69,70]. An example of the 2D system is shown in Figure S3 in Section S2. In other aspects, Gamma distribution could also be derived as the probability for at least α random events happening between time t and t+dt [68].

In summary, the Gamma distribution is strongly associated with the grain size in a Poisson–Voronoi nucleation/growth model. In other words, if the centers of grains at a given time are randomly distributed across the entire volume, the size distribution of the Voronoi cells generated from these points is expected to be fitted with Gamma distribution [71]. However, the Gamma distribution connected to electrodeposition is still unknown, and there could be other explanations for the occurrence of Gamma distribution.

### 3.5. Fitting with Gamma, Lognormal, and 2-Parameter Weibull Distributions

Before defining the parameters in the fitting functions, we should define the average value of the grain area and other properties. The mean grain size $R_{0}$ is averaged based on the radius of the grains ($R_{i}$), instead of the area of grains:

(5) $$R_{0}=\frac{\sum_{i}R_{i}}{N^{tot}}$$

All the fittings were conducted using the cumulative distribution function (CDF) of each distribution since the choice of bin size might impact the fitting results with the probability density functions (PDFs). For more details about the applications, PDFs, and physical pictures of the generalized Gamma distribution, the Lognormal distribution, the Weibull distribution, and the Gamma distribution, a short commentary is given in Section S2. An example of fitting the particle size with CDF is shown in Section S3.

Regarding the cumulative distribution function (CDF) of the Lognormal distribution for feature R, with two fitting parameters ($\gamma_{L}$, $k_{L}$), we have

(6) $$F\left(R\right)=\frac{N_{tot}}{2}\left(1+\mathrm{erf}\left(\frac{k_{L}\ln\left(R/\gamma_{L}R_{0}\right)}{\sqrt{2}}\right)\right)$$

Regarding the CDF of the Weibull distribution for feature R, with two fitting parameters ($\gamma_{W}$, $k_{W}$), we have

(7) $$F\left(R\right)=N_{tot}\left(1-\exp\left(-{\left(\frac{R}{\gamma_{W}R_{0}}\right)}^{k_{W}}\right)\right)$$

Regarding the CDF of the Gamma distribution for the feature $A_{R}$, with two fitting parameters ($\alpha^{\prime }$, $\gamma_{G}$), we have

(8) $$F\left(A_{R}\right)=\frac{N^{tot}}{\Gamma \left(\alpha^{\prime }\right)}{\left(\frac{\gamma_{G}}{A_{0}}\right)}^{\alpha^{\prime }}\int_{o}^{A_{R}}A_{\;}^{\alpha^{\prime }-1}\exp\left(-\frac{\gamma_{G}A}{A_{0}}\right)dA$$

Note that $R_{0}$ is the average radius of the grains, whereas $A_{0}$ is the average area of the grains. We will use their CDF functions to fit the radii derived from the observed areas (assumed to be circular particles) from the SEM images.

### 3.6. Behavior of the Fittings

By defining the fitting parameter γ based on the average values of the observed distributions for the Lognormal and Weibull distributions, we could expect γ→1. The mean values of Lognormal and Weibull distributions based on our definitions can be calculated as

(9) $${\bar{R}}_{L}=\gamma_{L}R_{0}\exp\left(\frac{1}{2k_{L}^{2}}\right)$$

(10) $${\bar{R}}_{W}=\gamma_{W}R_{0}\cdot \Gamma \left(1+\frac{1}{k_{W}}\right)$$

Since the fitted parameter k is large for both Lognormal and Weibull distributions, $\exp\left(\frac{1}{2k_{L}^{2}}\right)$ and $\Gamma \left(1+\frac{1}{k_{W}}\right)$ are closer to the value of 1. Successful fitting requires that the mean of the fitted distribution function be very close to the mean value of the original dataset ($\bar{R}\to R_{0}$). Therefore, the fitted γ parameters are very close to the value of 1.

Regarding the Gamma distribution, both fitting parameters have similar fitted values. Considering the mean value of Gamma distribution,

(11) $${\bar{A}}_{G}=\frac{\alpha^{\prime }A_{0}}{\gamma_{G}}$$

Since successful fitting requires the mean of the fitted function ${\bar{A}}_{G}$ be very close to the mean of the observed distribution, $\alpha^{\prime }\approx \gamma_{G}$ for successful fittings with Gamma distribution.

**Figure 2:**
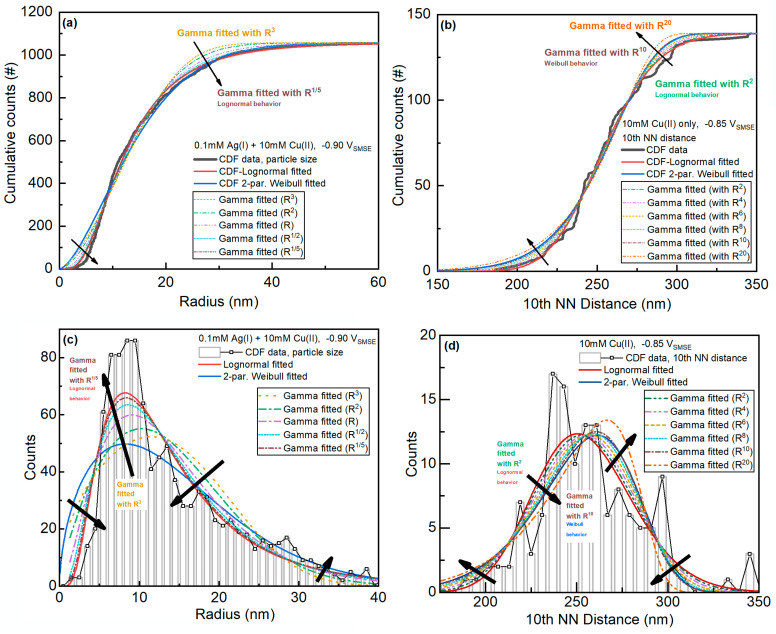
Comparison between Gamma fitting results from different definitions of grain sizes and Lognormal or Weibull fitting results (**a**–**d**). “#” means count numbers, and arrows means the general trend of Gamma distribution when defining the data in increasing dimensions, which at certain values can behave like the Weibull or the Lognormal distribution.

For some of the fitting results, the Gamma distribution fitting result seems to be in the middle of Lognormal and Weibull fitting results. The Gamma, Lognormal, and Weibull distributions are all derived from the generalized Gamma distributions. To examine the relationship between these distributions in the fittings, fittings of the distributions of particle size and nearest-neighbor distance in different orders ($R^{i}$) will be used as examples (Figure 2).

The data in Figure 2a can be fitted with the Lognormal distribution. By decreasing the order i in the definition of the particle size, the Gamma distribution behaves more like the Lognormal distribution fitting results. In other words, the fitting parameters of the Gamma distribution are very large (e.g., values of 12 or even 30), and the fitted distribution behaves like the Lognormal distribution.

The data in Figure 2b agree well with the Weibull distribution. By increasing the order i in the definition of the nearest-neighbor distance, the Gamma distribution behaves more like the Weibull distribution results (especially when $i\sim k_{W}$). This also means that if the fitted parameters of the Gamma distribution are very small (e.g., values of 2–4), the fitted distribution behaves similarly to the Weibull distribution. Thus, the Gamma distribution can behave completely differently from both distributions regardless of the definition of the dimension of the data.

## 4. Results and Discussions

### 4.1. Statistics of the Features

**Figure 3:**
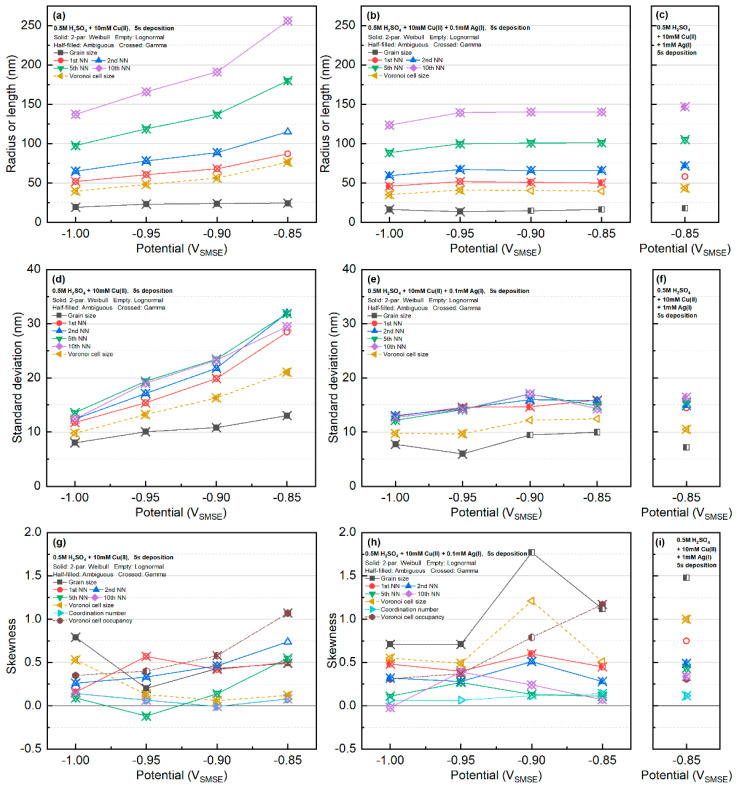
Summary of the statistics of the SEM images: (**a**–**c**) mean radius/length, (**d**–**f**) standard deviation, and (**g**–**i**) skewness. Note that the occupancy is based on the area fraction of the grains inside their Voronoi cells. The mean, standard deviation, and skewness of the grain and Voronoi cell sizes are evaluated based on their linear size, assuming the particles are circular ($R_{i}={\left(A_{i}/\pi \right)}^{0.5}$). Values in this figure are tabulated in Section S4.

The calculated mean and standard deviation of the particles in the SEM images (table in Section S4) are summarized in Figure 3. The mean value of all the features with the Cu-only systems significantly decreased with respect to the increase in the deposition overpotential. The decreasing nearest neighbor with respect to increasing deposition overpotential was also found in the Pb deposition at the vitreous carbon electrode [9]. The decreasing standard deviation of the Voronoi cell with increasing overpotential suggests that the microstructure of the Voronoi cells (related to the diffusion zones around each nucleus) becomes more regular [33].

On the other hand, when Ag was present, the average radius was nearly a constant above the applied potential of −1.00 V_SMSE_. The standard deviation of the features followed similar a trend. The standard deviations of the nearest neighbors were very close, regardless of the order of the nearest neighbor. There was no clear trend regarding the skewness of the data.

The standard deviations of the nearest-neighbor distances are shown in Figure 3b. Agreeing with the trend of the values with spatially completely random points, the standard deviations of the NN distances in Cu-only or Cu-Ag systems were nearly independent of the rank of the NN. Therefore, the different trends of the standard deviations of NN distances in Cu-only and Cu-Ag systems (Figure 3) were mainly caused by different nucleation densities at each deposition condition. Tsakova and Milchev observed that when normalizing the NN distances with average distance, the standard deviation decreases with increasing NN rank [18]. This behavior is caused by the difference in the mean distance at different NN ranks (Figure 3a).

### 4.2. Weibull, Lognormal, and Gamma Fitting Results

**Figure 4:**
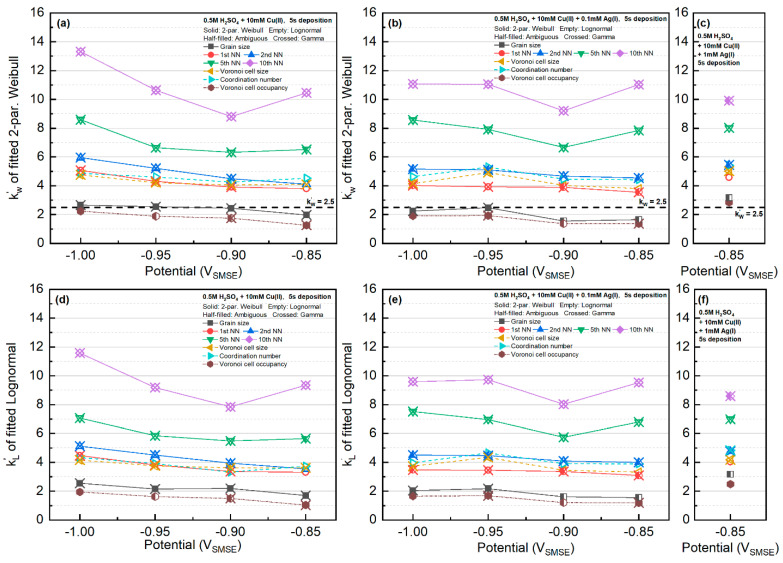
Results of the features fitted with Weibull distribution in $k_{W}^{\prime }$ parameter (**a**–**c**) and Lognormal distribution in $k_{L}^{\prime }$ parameter (**d**–**f**). The location of $k_{W}^{\prime }=2.5$ is marked in the Weibull distribution (**a**–**c**), whose value corresponds to the grain size statistics in a 2D steady-state grain growth simulation by Fayad et al. [41].

**Figure 5:**
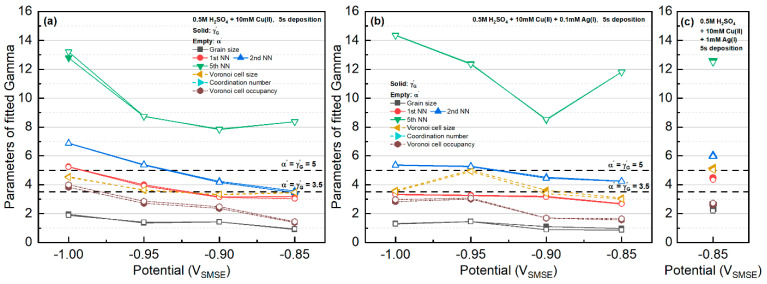
Fitting results of the Gamma distribution with both fitting parameters (**a**–**c**).

All the statistics were fitted with Lognormal, Weibull, and Gamma distributions. The fitting results for the Weibull distribution and the Lognormal distributions are presented in Figure 4, and the results for the Gamma distribution are shown in Figure 5. The fitted parameters and goodness of the fittings are tabulated in Section S5.

For the Cu-only electrolyte, the k fitting factors for Lognormal or Weibull distributions gradually increased with increasing deposition overpotential. The different trend of the 10th nearest-neighbor distribution was caused by the small sample size. When Ag was present in the electrolyte, except for the system at −0.90 V, the k factor was nearly independent of the applied potential. Particle size in most cases fulfilled Weibull distribution. Interestingly, the $k_{W}$ factor was very close to the value (~2.5) from the simulation of 2D grain growth by Fayad et al. [41].

The distribution of Voronoi cell size showed the opposite trend to that of the particle size. At small overpotentials, the Voronoi cell size distribution behaved like the Weibull distribution. However, at larger deposition overpotential, the Voronoi cell size behaved like the Lognormal distribution.

Considering its dependence on the radius and the k factor, the Voronoi cell size had roughly twice the value of the particle size. On the other hand, the k factors for the Voronoi cell size were very similar to the first nearest-neighbor distance and the coordination number of the grains.

Regarding the $\gamma_{W}$ and $\gamma_{L}$ fitting parameters, the value of $\gamma_{W}\approx 1$ for all features, whereas the $\gamma_{L}$ in the Lognormal distribution was in the range of 0.7~0.9. The potential dependence of $\gamma_{W}$ and $\gamma_{L}$ on different features is included in Figure 4.

The fitting results of the Gamma function with the features of the Cu-only and Cu-Ag deposits are shown in Figure 5. For both Cu-only and Cu-Ag systems, the parameters fitted with the Gamma function slightly increased with deposition overpotential. The increases in the fitting parameters with the Cu-only samples were more significant than those of the Cu-Ag systems. The two fitted parameters of the Gamma distribution ($\gamma_{G}$ and $\alpha^{\prime }$) had very similar values for all the features investigated. Such behavior indicates that the mean value of the actual data is a good estimation of the mean value of the fitted Gamma distribution.

In contrast to the Weibull and Lognormal distribution fitting, the fitting parameters of the Gamma distribution significantly varied with respect to the definition of the particle shape. The fitted Gamma distribution can behave like a Lognormal or Weibull distribution if the dimension of the data are defined appropriately (Figure 2). The Gamma distribution fittings of the particle size and the Voronoi cell size behaved like the Weibull distribution, whereas the Gamma distribution fittings of the n-th rank nearest-neighbor distances behaved like the Lognormal distribution.

### 4.3. Particle Sizes, Voronoi Cell, and Coordination Number

**Figure 6:**
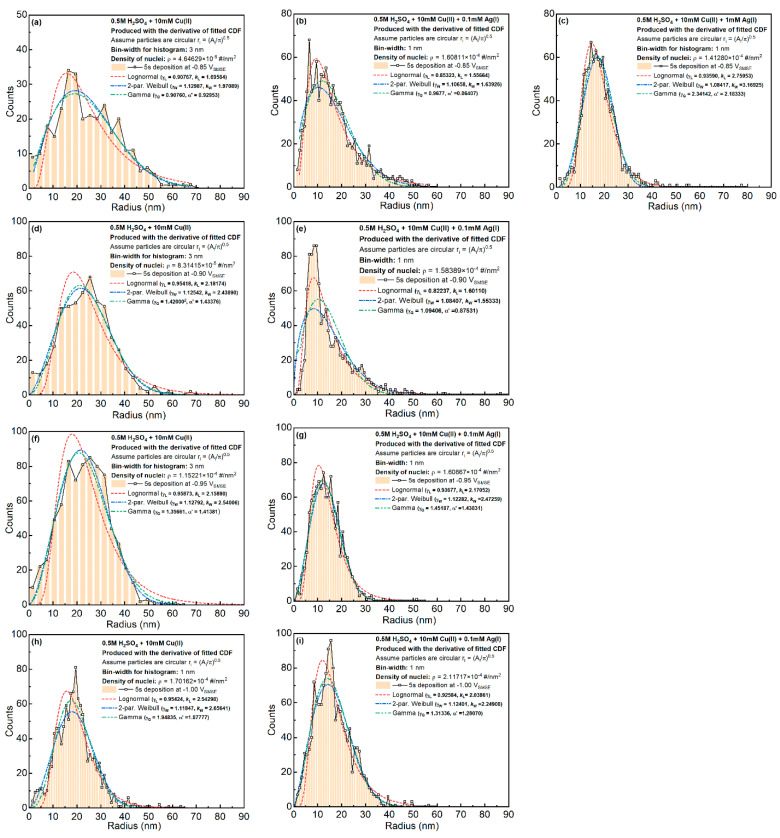
Particle size distributions for all the Cu-only and Cu-Ag samples. Different columns correspond to different Ag concentrations and different rows represent different deposition potentials (**a**–**i**). “#” in the labels mean the number of counts.

The particle size distributions are shown in Figure 6. The fluctuations in the plot were caused by the small bin size used for calculating the distributions. The particle size distribution was highly skewed towards the lower radius direction. When more Ag was added to the system, the average and the variation in the particle size became smaller. For most of the samples, Weibull or Gamma fitting could describe the general shape of the CDF curves. Some of the samples (Figure 6b,c,e) possessed a trend between the Lognormal and the Weibull distribution.

**Figure 7:**
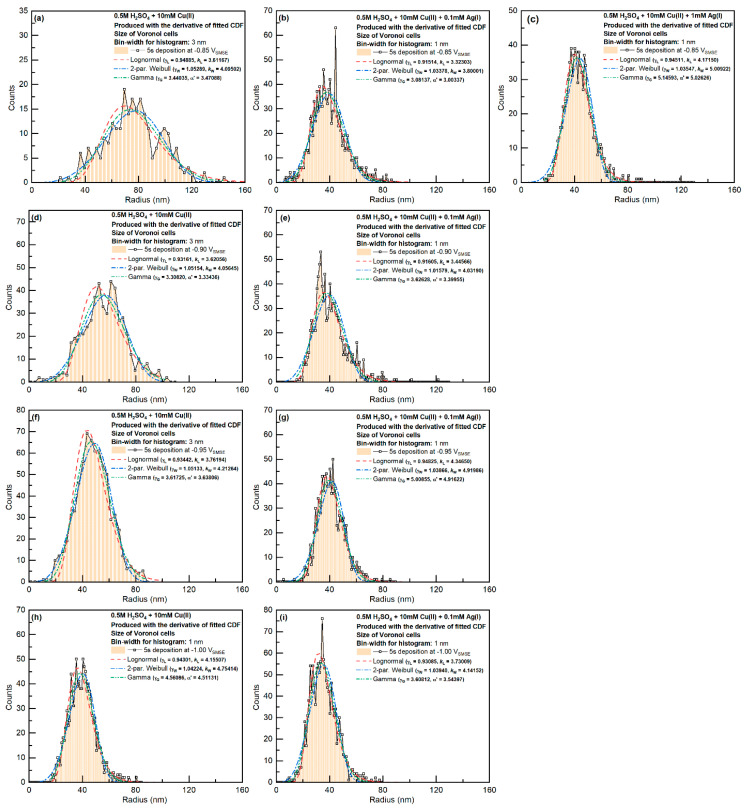
Voronoi cell size distributions for all Cu-only and Cu-Ag samples. The average radius was calculated based on the average area, assuming a circular shape (instead of the average of the linear dimensions of the Voronoi cells). Different columns correspond to different Ag concentrations (**a**–**i**).

The distributions of the Voronoi cell sizes are shown in Figure 7. Note that the Voronoi cells were generated based on the boundary of the grains, instead of their weight center. For Cu-only systems with a −0.85 V_SMSE_ deposition potential, the Voronoi cell sizes could be fitted with the Weibull distribution. When Ag(I) was added to the system or the deposition overpotential increased, the Voronoi cell sizes behaved more similarly to the Lognormal distribution.

**Figure 8:**
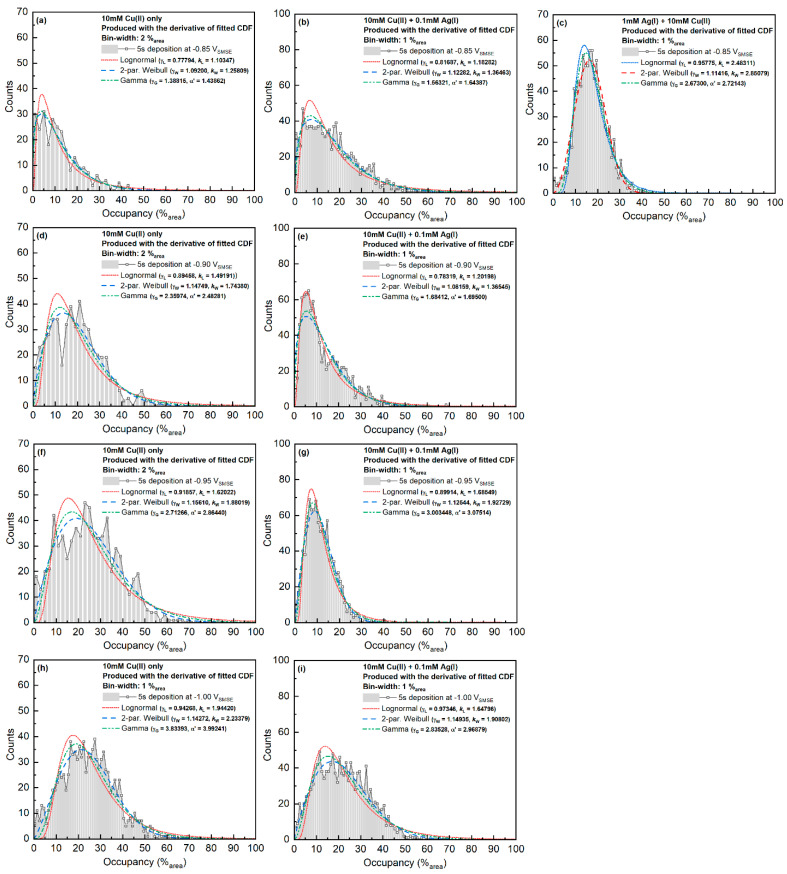
Voronoi cell area occupancy. Different columns correspond to different Ag concentrations, and different rows represent different deposition potentials (**a**–**i**).

Voronoi cell occupancy was defined by dividing the area of the deposit in the SEM image by the area of its Voronoi cell, with unit %_area_. The statistics of the Voronoi cell occupancy are shown in Figure 8. The higher the deposition overpotential, the wider the distribution becomes. Considering the nucleation density, the incomplete coalescence of diffusion zones in Cu-only systems leads to potential dependence on occupancy. However, a similar behavior was also observed in Cu-Ag samples, in which the nucleation densities were very similar. The shift of distribution in Cu-Ag samples might indicate the faster relative expansion rate of nucleation exclusion zones (compared with the diffusion zones) at higher potentials.

The mean and standard deviation of the Voronoi cell occupancy and the coordination number were also measured (Section S6). The mean occupancy of the Voronoi cells was between 10 and 30%_area_. The coordination number of the systems deposited below −0.85 V_SMSE_ showed a value of around 5.95, whereas the deposits at −0.85 V_SMSE_ showed a value of around 6.00. This number is very close to the expected value of 6 from Euler’s law [22]. The nucleation and growth of a single nucleus are mostly influenced by the diffusion fields from the six nearest neighbors on average. Such behavior could explain why the NN distances from experiments agree well with the simulated results only when considering the concentration fields from fewer than the five nearest neighbors [13]. The coordination number for most of the samples fulfilled Lognormal distribution, whereas the area occupancy of each Voronoi cell fulfilled Weibull distribution in most cases.

### 4.4. Nearest-Neighbor Distance and Radial Distribution Function

**Figure 9:**
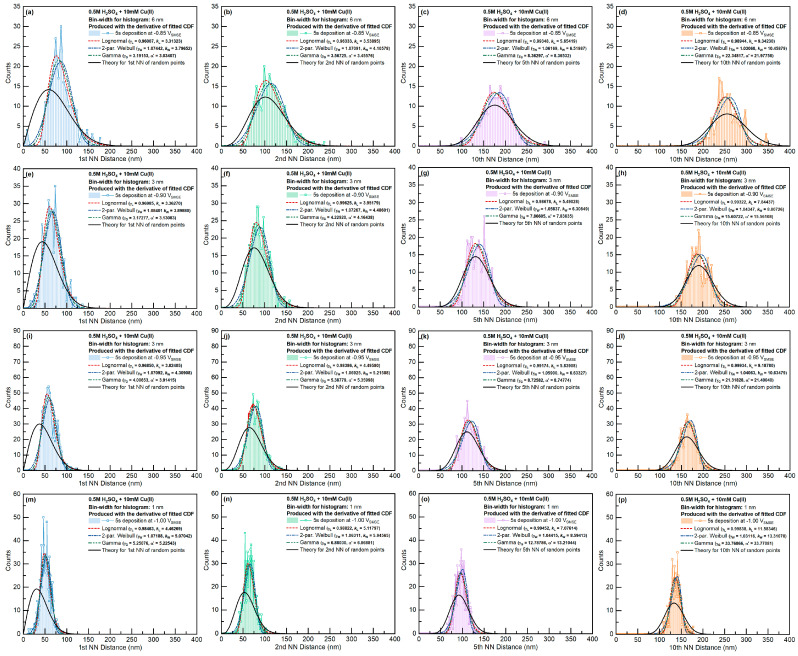
NN distance distribution for 10 mM Cu(II) at different deposition potentials. Image size: 3000 × 2250 nm^2^. Different columns represent different orders of nearest neighbors, and different rows represent different applied potentials (**a**–**p**).

**Figure 10:**
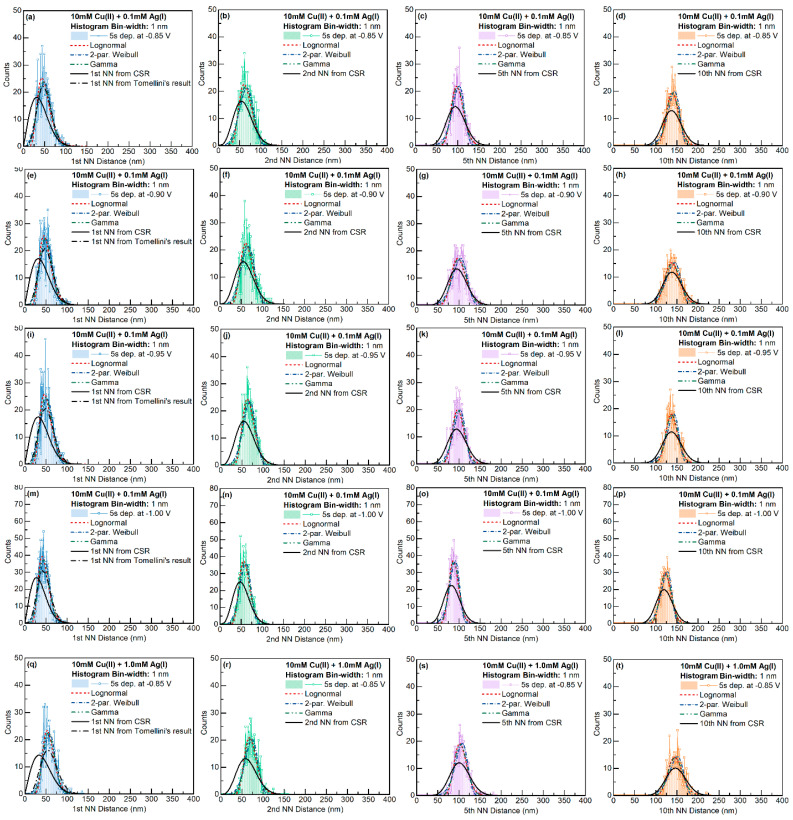
NN distance distribution for 10 mM Cu(II) + 0.1 or 1 mM Ag(I) at different deposition potentials. Image size: 3000 × 2250 nm^2^. Different columns represent different orders of nearest neighbors, and different rows represent different applied potentials (**a**–**t**).

The n-th nearest-neighbor distance was evaluated with all nuclei in the imaging area. The closest neighbor distances of the system were evaluated based on the mass center of the grains. To avoid the influence from the edge of the imaging area, only the particles with a distance larger than the maximum distance of each rank of the nearest neighbor from the edges were counted. The distributions of the 1st, 2nd, 5th, and 10th nearest neighbors for the Cu-only and Cu-Ag systems are given in Figure 9 and Figure 10, respectively.

To evaluate the impact of the nucleation exclusion effect, the observed distributions were compared with analytical results for the NN distances with complete spatial randomness (Equation (2)). As expected, exclusion zones occurred in the distributions of the first and second nearest neighbors. The exclusion between particles could be caused by (1) the nucleation exclusion zones around the early-formed nuclei or (2) the size of the particles (since we used the mass center of the particles to evaluate nearest-neighbor distances). At higher-rank nearest neighbors, the exclusion effect between particles was weakened. The fitted distributions had smaller variations compared with the model based on completely spatially random points.

The observed first NN distance distributions were further compared with Tomellini’s model, which considers the nucleation exclusion effect when nuclei are formed at different times. As mentioned in Section 3.1, Tomellini’s model only introduced two of the three fitting parameters; this model does not consider how to calculate the mean first NN distance based on a certain definition of nucleation exclusion zone with parameters from the mass transport and nucleation kinetics of actual electrodeposition. To compare the shape of Tomellini’s model with the observed distribution, we derived the fitting parameter γ from the average feature size $\mu_{act}$:

(12) $$\mu_{act}=\frac{\Gamma \left(\eta +\frac{1}{n}\right)}{(a{\rho )}^{1/n}\Gamma \left(\eta \right)}=\frac{1.69626}{(a{\rho )}^{0.556}}$$

With n≈1.8, η≈2.8, and ${\left(1.70/\mu_{act}\right)}^{1.8}\to \alpha \rho$, we could compare the observed distribution with Tomellini’s result using Equation (2). As seen in Figure 9 and Figure 10, for all the samples, the actual distributions had a smaller standard deviation compared with the results from Tomellini’s model. Such behavior suggests that the actual nucleation exclusion zone was smaller than the size assumed in the diffusion zone problem for the particle with a large nucleation exclusion zone, and vice versa. At lower deposition potentials, Tomellini’s model agrees well with the observed distributions of the first NN distances.

**Figure 11:**
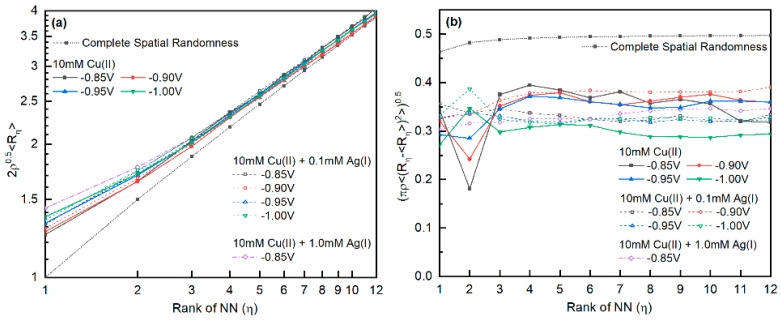
(**a**) Log–log plot of the mean distance of the pair of nuclei neighbor and (**b**) standard deviations of the 1–12 ranks of nearest-neighbor distances. Both values were normalized based on the nucleation density ρ (instead of average distance). Values corresponding to the nearest-neighbor distance of spatially completely random points are drawn as reference lines.

The relationship between $2\rho^{0.5}\langle R_{\eta }\rangle$ and the rank η of the nearest neighbor is shown in Figure 11a [33,72]. Compared with that of spatially completely random points, a larger mean NN distance between nuclei was observed in the Cu-Ag and Cu deposits, suggesting the impact of the nucleation exclusion zones. Interestingly, there was no significant difference between the curves with or without Ag(I). Therefore, the nucleation and growth of both Ag and Cu, either under progressive or instantaneous nucleation kinetics, were influenced by the nucleation exclusion zones. For the Cu-only system, the randomness of the nucleus improved with decreasing deposition overpotential, agreeing with the statement by Serruya et al. [10]. However, when Ag(I) was added, the systems did not have a clear trend between spatial randomness and the applied potential.

The spatial randomness of the particles was further evaluated with the radial distribution function (RDF) of the mass centers. To avoid the impact of the boundaries, only points at least 500 nm away from the edges of the imaging area were chosen. The RDF $g\left(r\right)$ was calculated from

(13) $$g\left(r\right)=\frac{N\left(r+\Delta r/2\right)-N\left(r-\Delta r/2\right)}{2\pi r\cdot \Delta r\cdot N_{ct}\;\rho_{nu}}$$

Note that $N_{ct}$ is the number of nuclei used for the counting process, $N\left(r\right)$ is the cumulative distribution of neighbors at distance r for all $N_{ct}$ nuclei, $\rho_{nu}=N^{tot}/V^{tot}$ is the nucleation density, and r is the radius away from one nucleus. The value of $N\left(r+\Delta r/2\right)-N\left(r-\Delta r/2\right)$ could be directly obtained by constructing a histogram with Δr as the bin size. To compare the effect of the nucleation exclusion zone of samples with different mean grain sizes, the NN distance $R_{NN}$ was normalized based on the average grain radius ($r=R_{NN}/\langle 2R_{grain}\rangle$).

**Figure 12:**
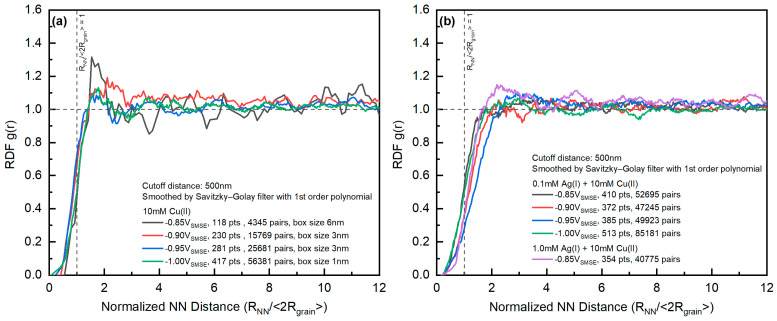
RDF of the (**a**) Cu-only samples and (**b**) Cu-Ag samples at various deposition potentials. Vertical dotted lines are the average diameter of the grains. The horizontal dashed line is the radial distribution function with complete spatial randomness.

A weak peak could be observed at the edge of the RDFs (Figure 12), indicating the impact of nucleation exclusion effects on the spatial distribution of the grains. There was no significant trend between the RDFs from different deposition conditions, considering the noise level in the RDFs. The edges of the Cu-only systems were closer to the average diameter of the grains, indicating that the nucleation exclusion had a stronger effect on the nucleation behavior of the Cu-Ag systems. In contrast, the RDF of the Cu-Ag system gradually deviated from the average diameter of grains with increasing deposition overpotential (above deposition potential of −1.00 V_SMSE_).

### 4.5. Grain Size Distribution from Features of Voronoi Cells

Note that the grain size distribution was fitted and discussed in Section 4.3 of this work. This section mainly discusses the grain size distribution derived from the Voronoi cell features.

Since the Voronoi cell occupancy is defined according to the area fraction of grains in each Voronoi cell, by assuming the partition of each Voronoi cell is independent of surrounding cells, we could derive the grain size distribution from the distributions of Voronoi cell size and occupancy by simply generating the histogram of each observed Voronoi cell size multiplied by each Voronoi cell occupancy.

**Figure 13:**
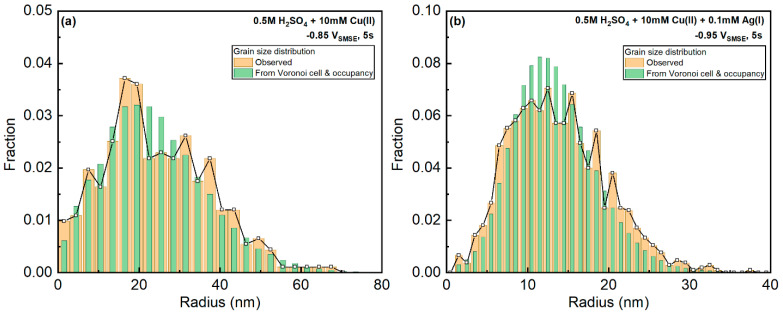
Comparison between observed and estimated (from Voronoi cell size and occupancy) grain sizes. (**a**) Cu-only deposits from 10 mM Cu(II) electrolyte at −0.85 V_SMSE_ for 5 s. (**b**) Cu-Ag deposits from 10 mM Cu(II) + 0.1 mM Ag(I) electrolyte at −0.95 V_SMSE_ for 5 s.

The observed and estimated grain size distributions are compared in Figure 13a,b. The Cu-only sample, at −0.85 V_SMSE_, showed a good agreement between the estimated and the observed distributions. However, the Ag-Cu sample, at −0.95 V_SMSE_, revealed that the predicted distribution was narrower than the observed one. Such behavior suggests that larger Voronoi cells tend to have a larger occupation fraction and smaller cells have a smaller occupation. This is reasonable considering that the smaller nuclei are later formed and influenced by the diffusion fields from surrounding nuclei. Therefore, the diffusion fields from surrounding grains have less impact on the grain growth of Cu-only systems than Cu-Ag systems.

### 4.6. Spatial Distribution of Nuclei under Instantaneous and Progressive Nucleation Modes

In the literature, the instantaneous nucleation mode is always simulated with spatially completely random points. However, features of the Cu-Ag deposits imply that nucleation exclusion behavior is more significant in Cu-Ag systems than Cu-only systems.

After examining the details in the classical model for the potentiostatic transients during electrodeposition, we found that, when following the assumptions of nucleation kinetics in the S-H model (the nucleation rate at the free area is proportional to the fraction of the nucleation-exclusion-zone-free area), the instantaneous nucleation mode must be preceded by a progressive nucleation stage. Following this statement, if the nucleation and growth in the electrodeposition system are only governed by the behavior of ions, the nuclei density for the instantaneous stage should also show an overpotential dependence due to the progressive nucleation stage. Such behavior was not observed in our Cu-Ag deposits. Thus, the nucleation kinetics of the Cu-Ag samples (instantaneous mode) is not governed by the kinetics related to the Ag(I)/Ag deposition step. We hypothesize that the saturation nucleation density in those samples reflects the density of features (available nucleation sites) on the n-Si(001) substrate.

Regarding the spatial distribution of the nuclei, if the features at the substrate are completely spatially random, there will not be a progressive nucleation region in the potentiostatic transient, and the spatial distribution of the nucleus will be spatially random. The spatial inhomogeneity observed in the Cu-Ag samples thus reflects the spatial inhomogeneity of the available nucleation sites on the substrate.

## 5. Conclusions

In this work, we examined the grain statistics and spatial distribution of Cu and Cu-Ag electrodeposits from acidic sulfate baths at a fixed deposition cutoff time (5 s) and different deposition potentials. The features of the grains could be fitted with Lognormal, Weibull, and Gamma distributions. The grain size and Voronoi cell occupancy distributions could be better described by the Weibull distribution. The nearest-neighbor distances, Voronoi cell size, and grain coordination number could be better fitted with Gamma or Lognormal distributions. For the reader’s reference, the fitting parameters were listed and compared.

Judging from SEM images, at a given potential range and deposition cutoff time, the distribution of features of Cu-only systems depends strongly on the applied potential, whereas those of Cu-Ag are almost independent of the deposition potential. Thus, the Cu-only system is controlled by progressive nucleation of Cu, whereas the Cu-Ag system is controlled by the instantaneous nucleation of Ag. At a low deposition overpotential, the Ag nuclei serve as reduction centers for Cu(II).

Surprisingly, the Cu-Ag system, seemingly in order to follow instantaneous nucleation kinetics, is more severely impacted by the nucleation exclusion zones. By examining the details in the classical model for the potentiostatic transient, if the deposition is controlled by the reduction of ions (instead of substrate properties), all instantaneous nucleation modes should be preceded by a progressive nucleation stage. However, this behavior does not agree with the overpotential independence of the nucleation densities in the Cu-Ag systems. Thus, the saturation nucleation density of the Cu-Ag system is related to the nucleation site density of the substrate, and the distribution of Cu-Ag grains reflects the spatial inhomogeneity of these features on the substrate.

## Data Availability

The data is contained in the Supplementary Material of this paper.

## Supplementary Materials

The following supporting information can be downloaded at: https://www.mdpi.com/article/10.3390/ma16196452/s1.
