# Supplementary material for: Observation of Weibull, Lognormal, and Gamma Distributions in Electrodeposited Cu and Cu-Ag Particles
Source: Materials (Basel). 2023 Sep 28;16(19):6452. doi: 10.3390/ma16196452 (PMC10573702; doi:10.3390/ma16196452)
Supplement: Supplementary file 1 [file materials-16-06452-s001.zip › materials-2592475-supplementary.pdf]

# Supplemental Material

# Observation of Weibull, Lognormal, and Gamma distributions in electrodeposited Cu and Cu-Ag particles

Yunkai Sun[a*], Giovanni Zangari[a]

a. Department of Materials Science and Engineering, University of Virginia, Charlottesville, VA, 22904, USA

* Corresponding authors. Yúnkāi Sūn, Email: ys3ej@virginia.edu; Giovanni Zangari, Email: gz3e@virginia.edu

## Section S1. SEM images and Voronoi cells of the grains

**1mM Ag(I) + 10mM Cu(II), -0.85 $V_{SHE}$, 5s**

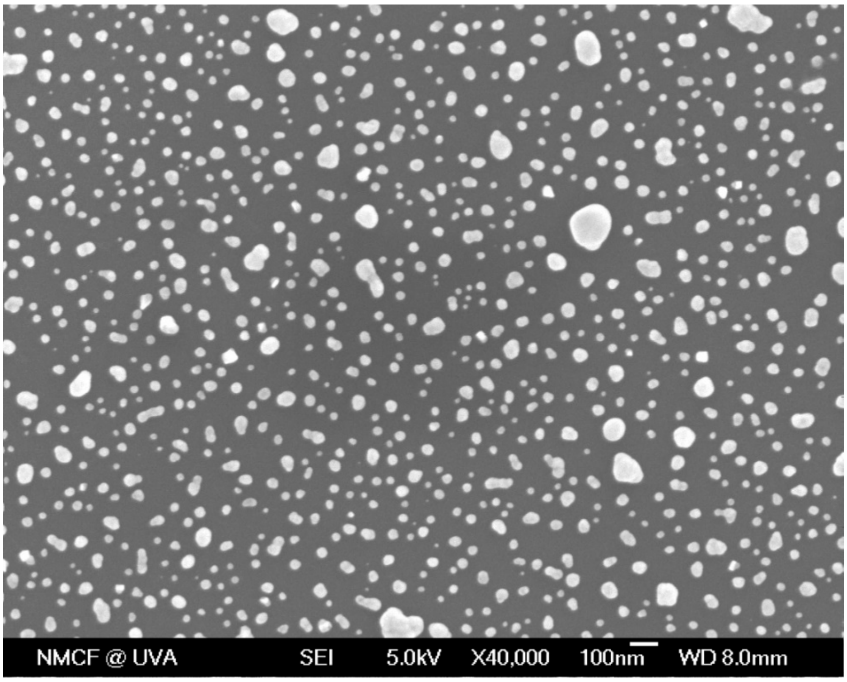


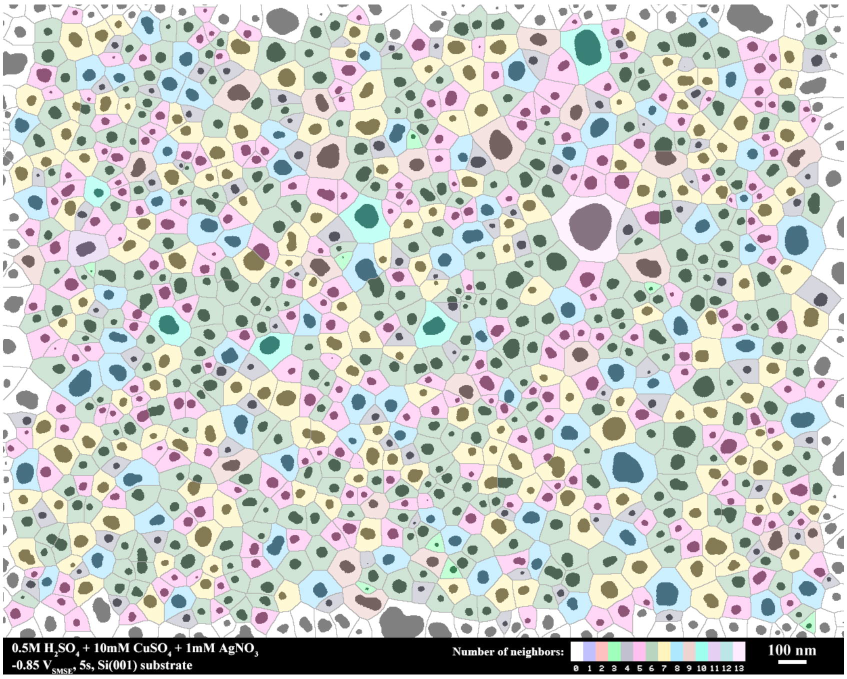

**0.1mM Ag(I) + 10mM Cu(II), -0.85 $V_{SHE}$, 5s**

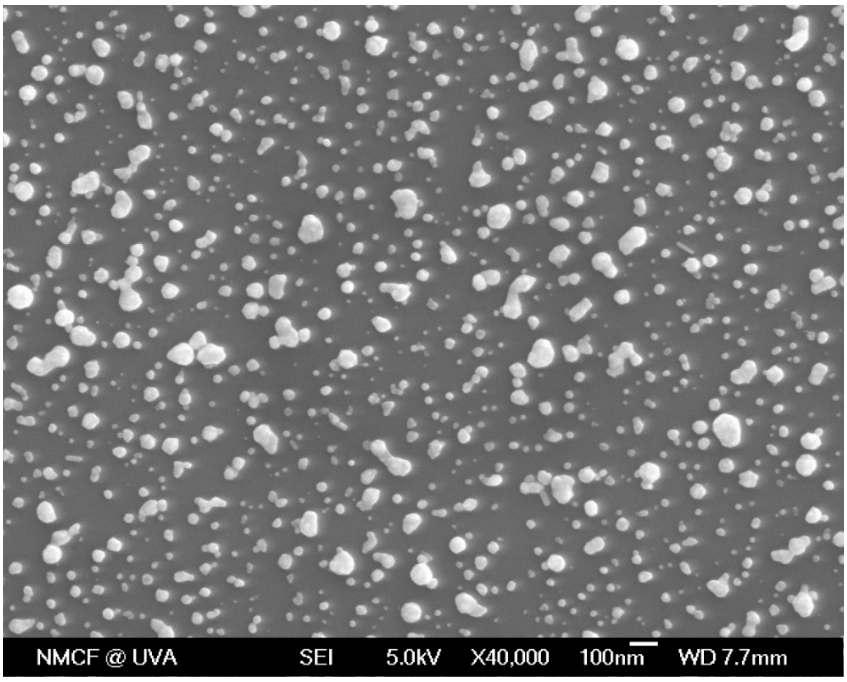


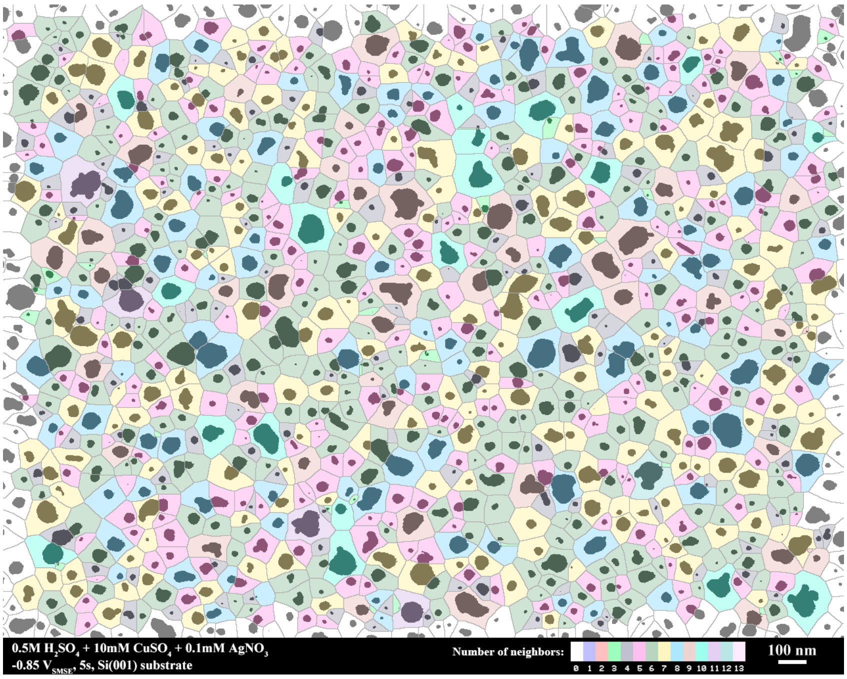

**0.1mM Ag(I) + 10mM Cu(II), -0.90 $V_{SHE}$, 5s**

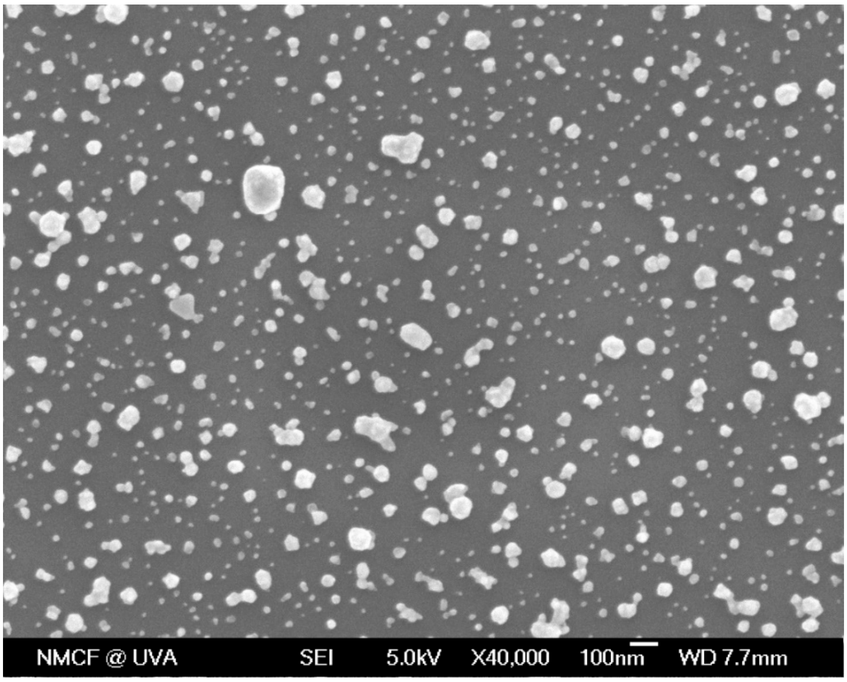


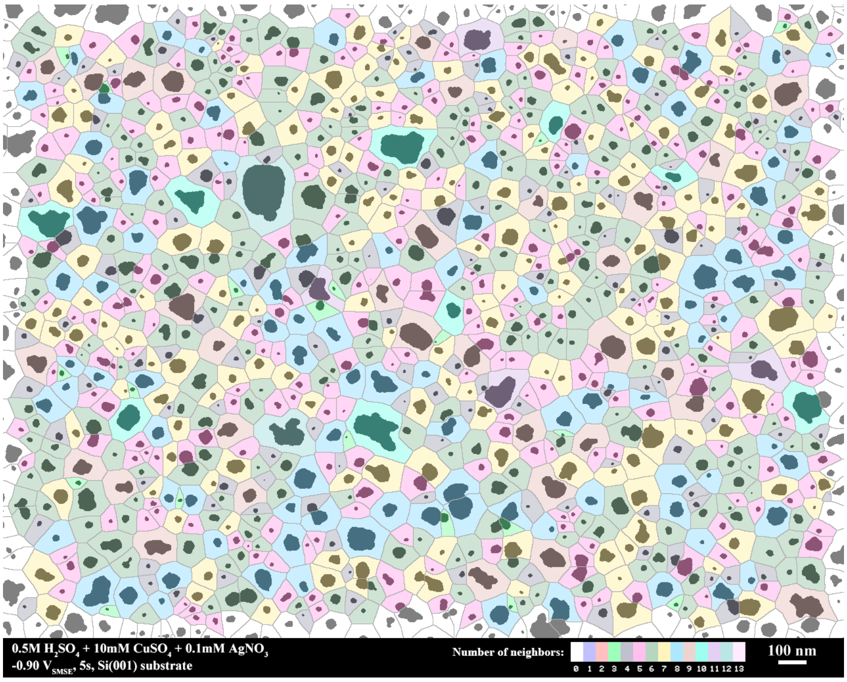

**0.1mM Ag(I) + 10mM Cu(II), -0.95 $V_{SHE}$, 5s**

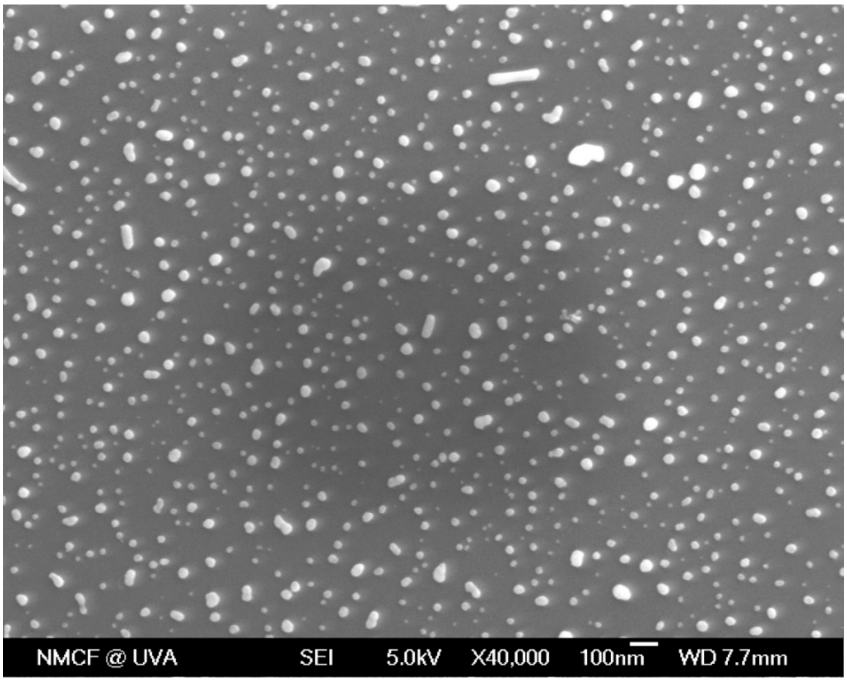


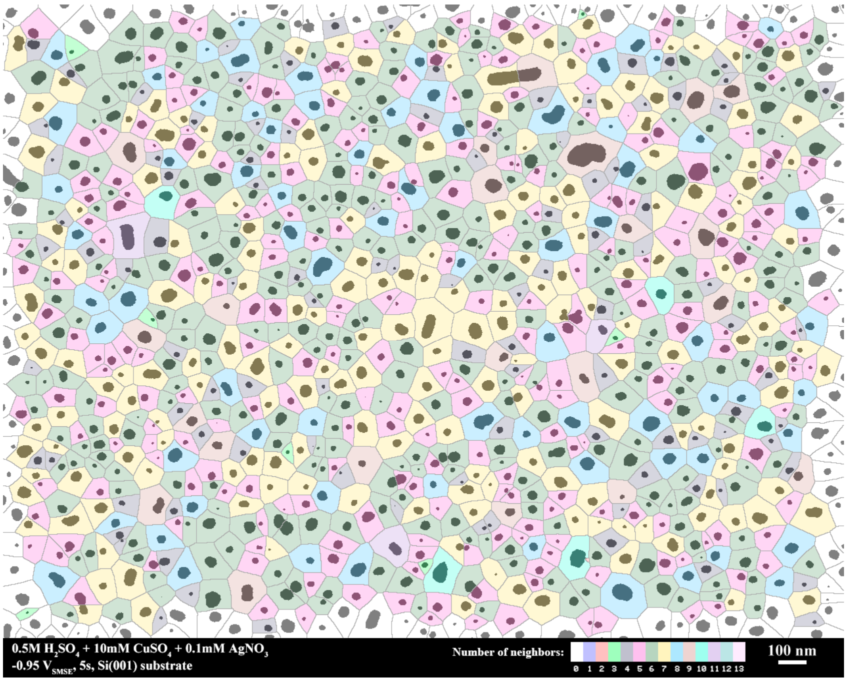

**0.1mM Ag(I) + 10mM Cu(II), -1.00 $V_{SHE}$, 5s**

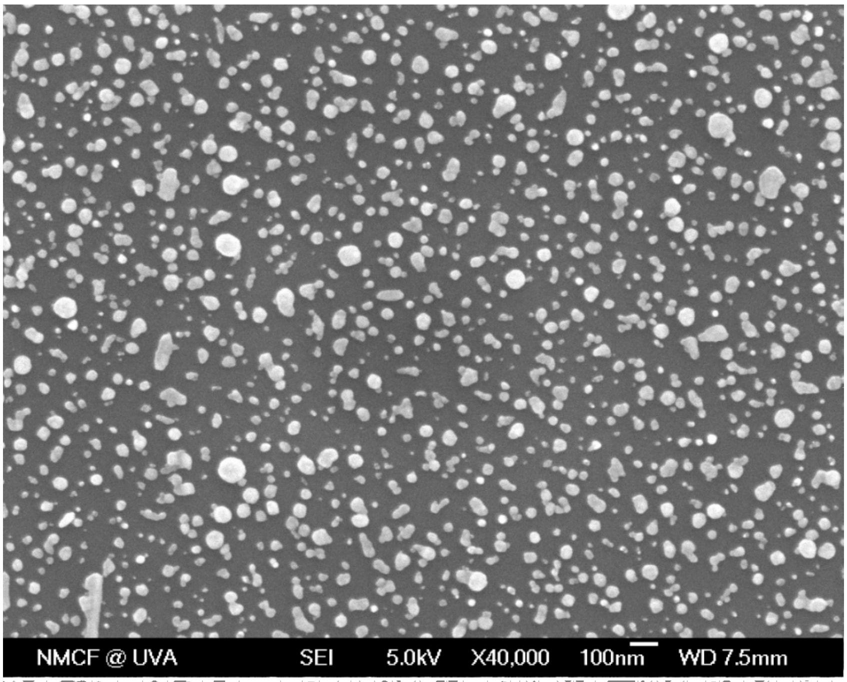


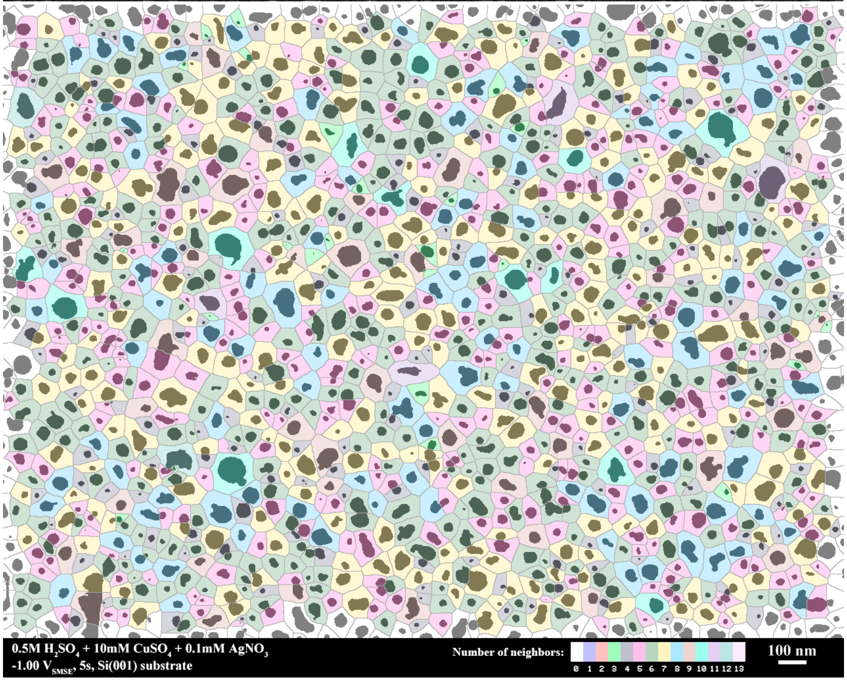

**10mM Cu(II), -0.85 $V_{SHE}$, 5s**

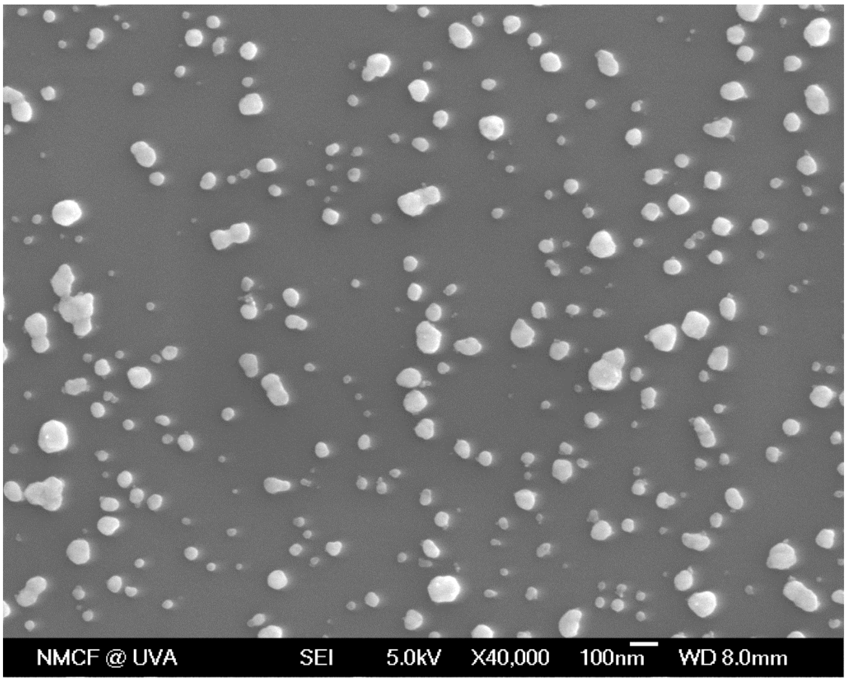


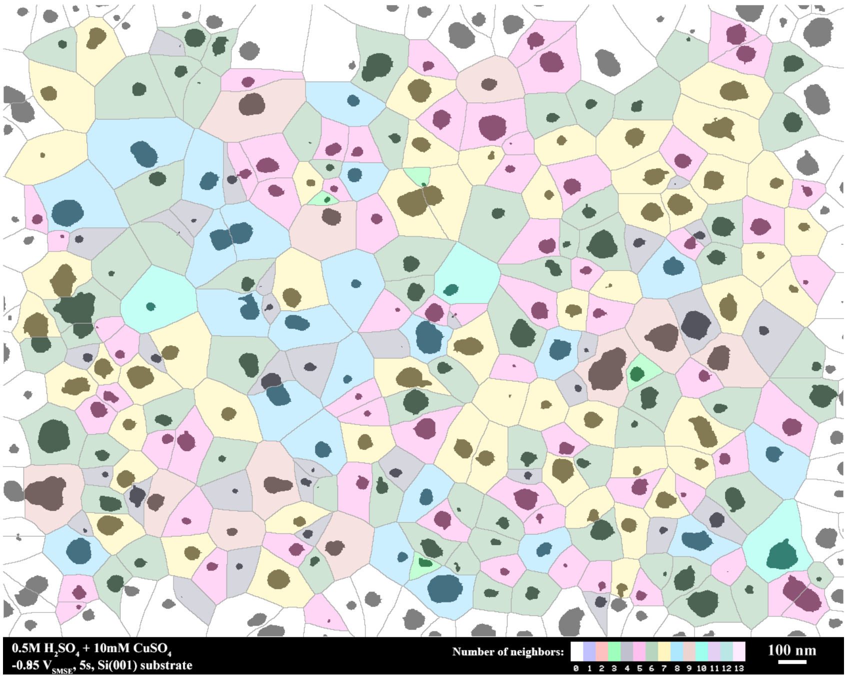

**10mM Cu(II), -0.90 $V_{SHE}$, 5s**

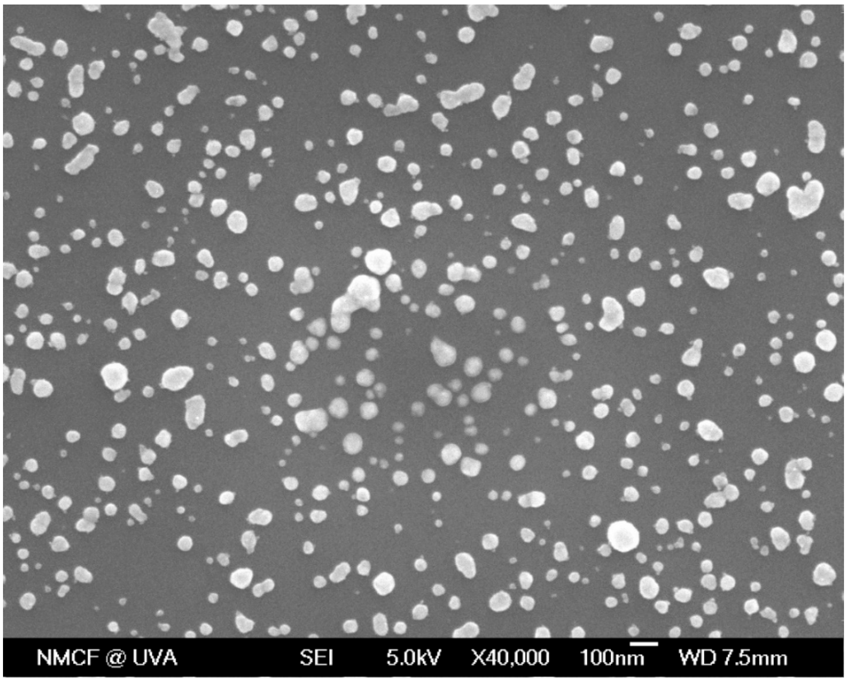


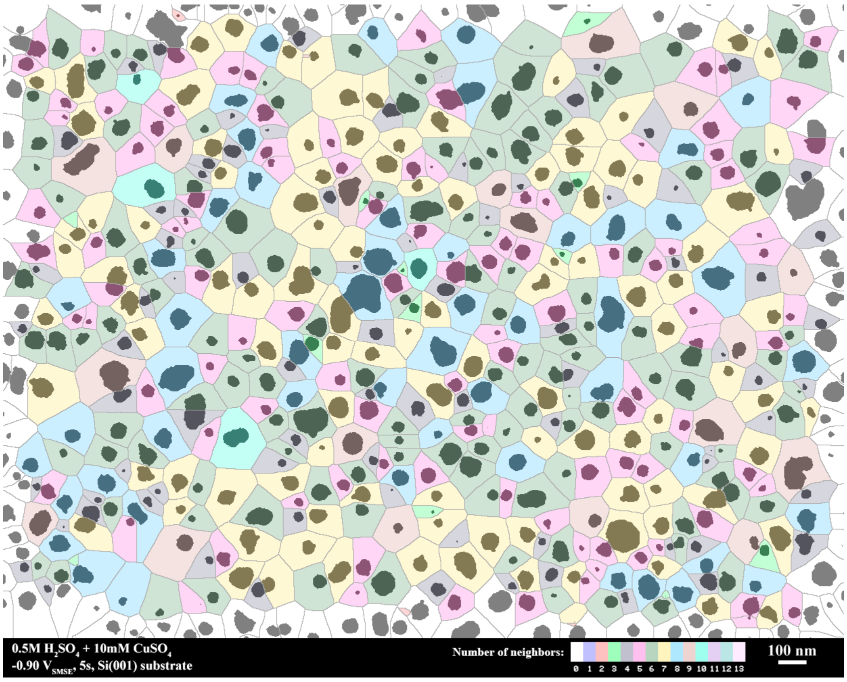

**10mM Cu(II), -0.95 $V_{SHE}$, 5s**

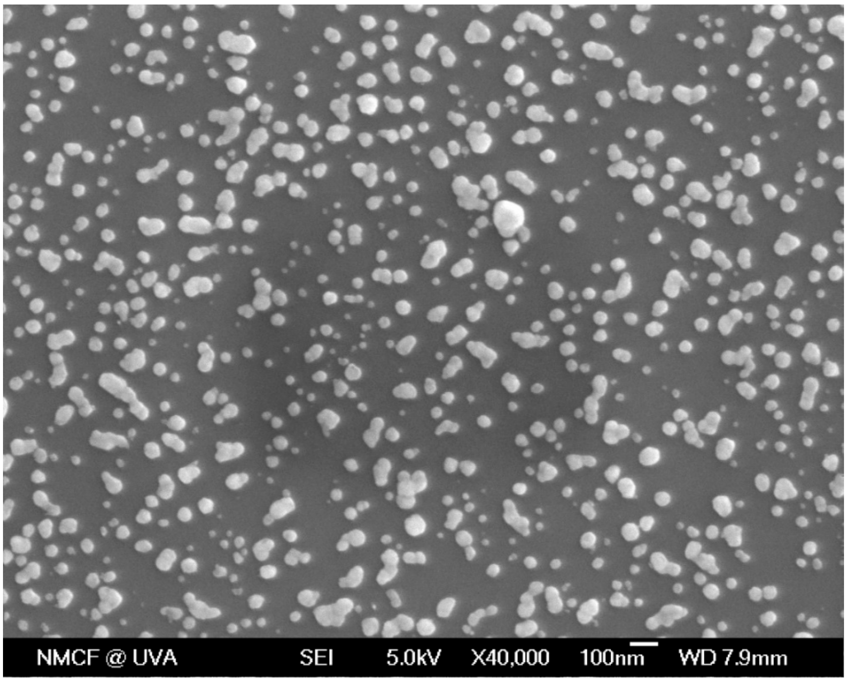


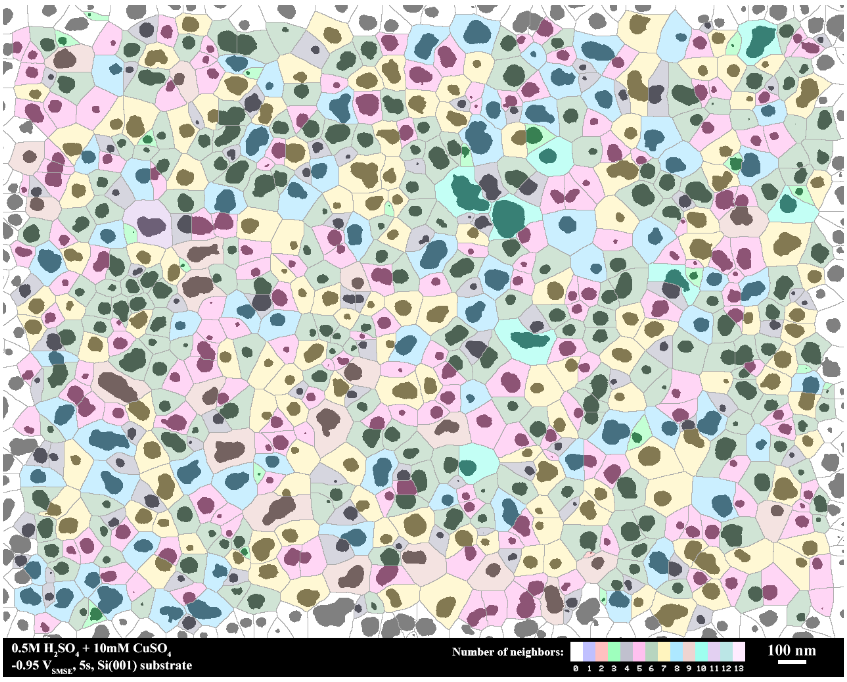

**10mM Cu(II), -1.00 $V_{SHE}$, 5s**

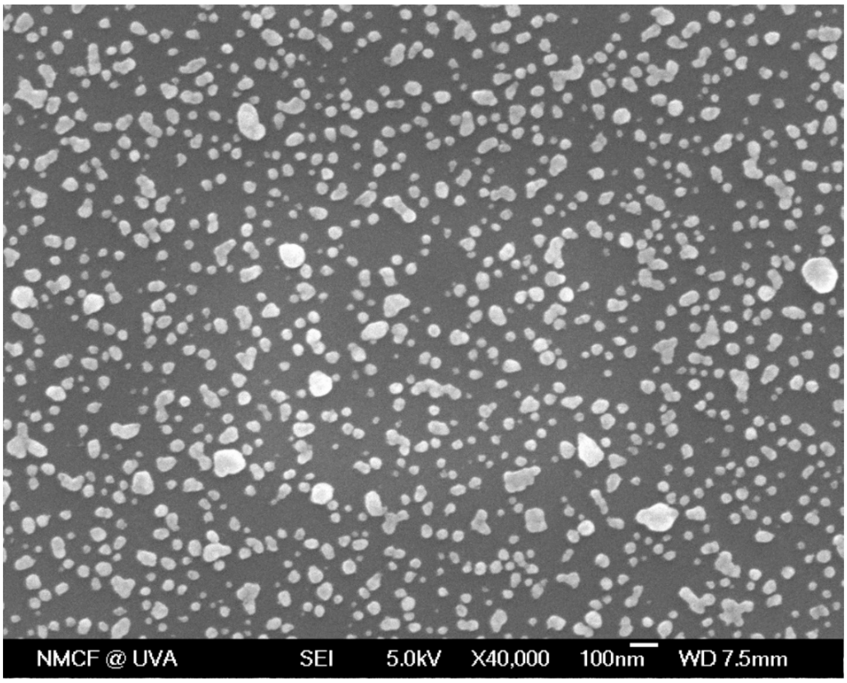


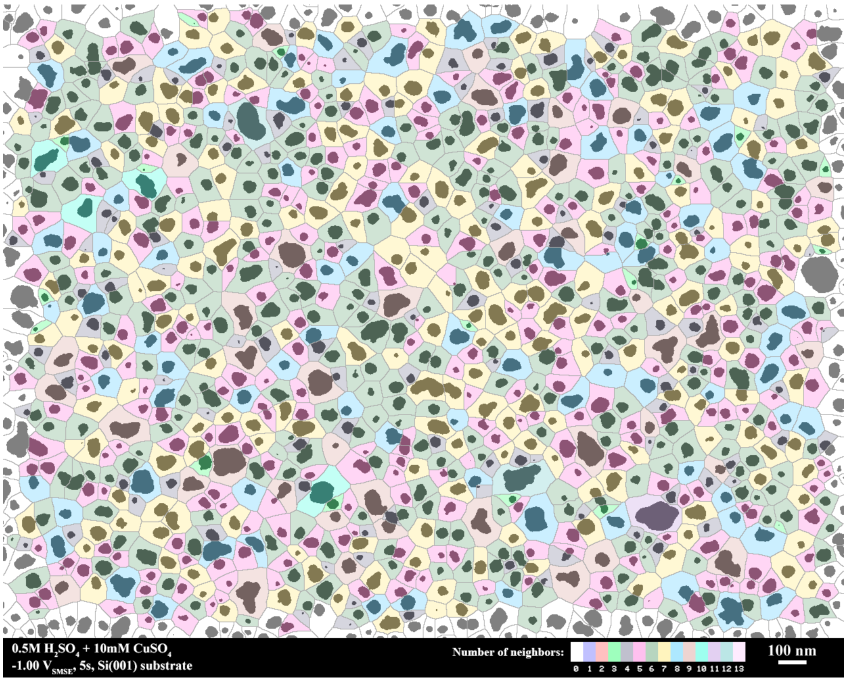

**Section S2 Comments on the Generalized Gamma distribution, the Lognormal distribution, the Weibull distribution, and the Gamma distribution**

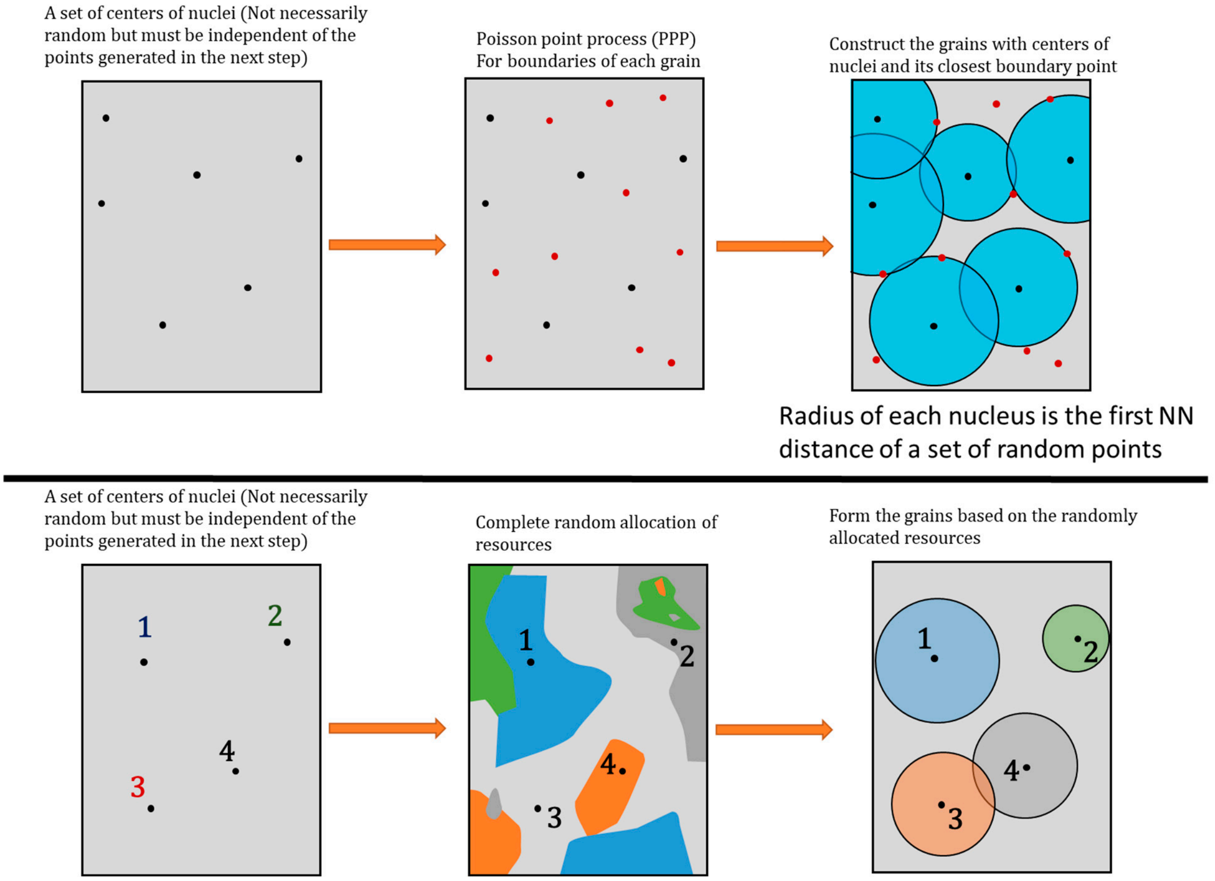


**Figure S1** Generation of Weibull distribution. Top figure indicates the random radius scenario, which should fulfill $k = 2$. Bottom figures indicate the random allocation of resources scenario, which should also fulfill $k = 2$.

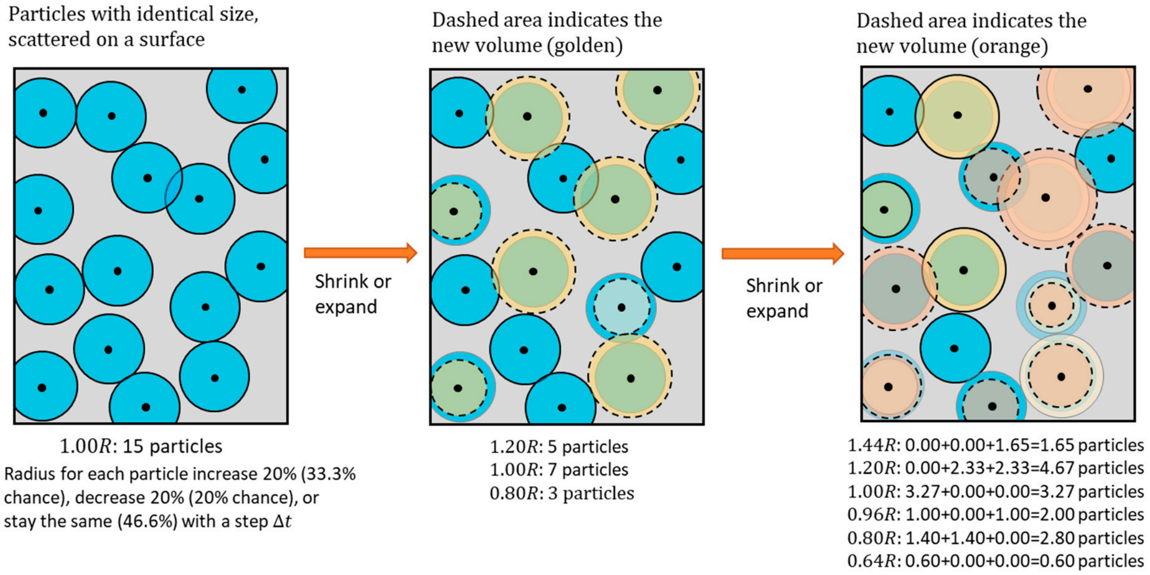


**Figure S2** Illustration of the law of proportionate with a hypothetical scenario of 2D overlapping circles, and random but time-independent growth rate. At the long-time limit, the distribution of these circles should fulfill the Lognormal distribution, with moments related to the randomness of the grow/shrink operation. At each step, $\langle \ln T \rangle = 0.333 \times \ln 1.2 + 0.2 \times \ln 0.8 = 0.016 > 0$. Therefore, the average radius of the particles (and thus the total area of the particles) will gradually increase. The dispersion of each growth step is $\langle (\ln T - \langle \ln T \rangle)^2 \rangle = 0.021$. When the step $n$ is large, this system should fulfill lognormal distribution with $\mu = n\langle \ln T \rangle = 0.016n$ and $\sigma^2 = n\langle (\ln T - \langle \ln T \rangle)^2 \rangle = 0.021n$. Details in **Appendix-A**.

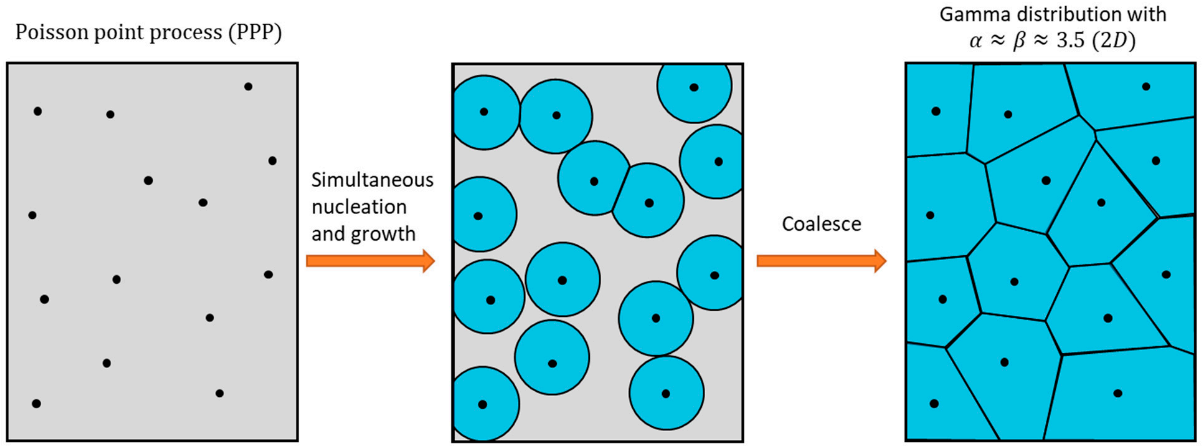


**<u>Figure S3</u>** Illustration on one of the scenarios where Gamma distribution could represent the final grain size distribution. The empirical results for a 2D complete spatially random nuclei is a Gamma distribution of the area of the cells with $\alpha \approx \beta \approx 3.5$.

## Section S3 Comments on the Generalized Gamma distribution, the Lognormal distribution, the Weibull distribution, and the Gamma distribution

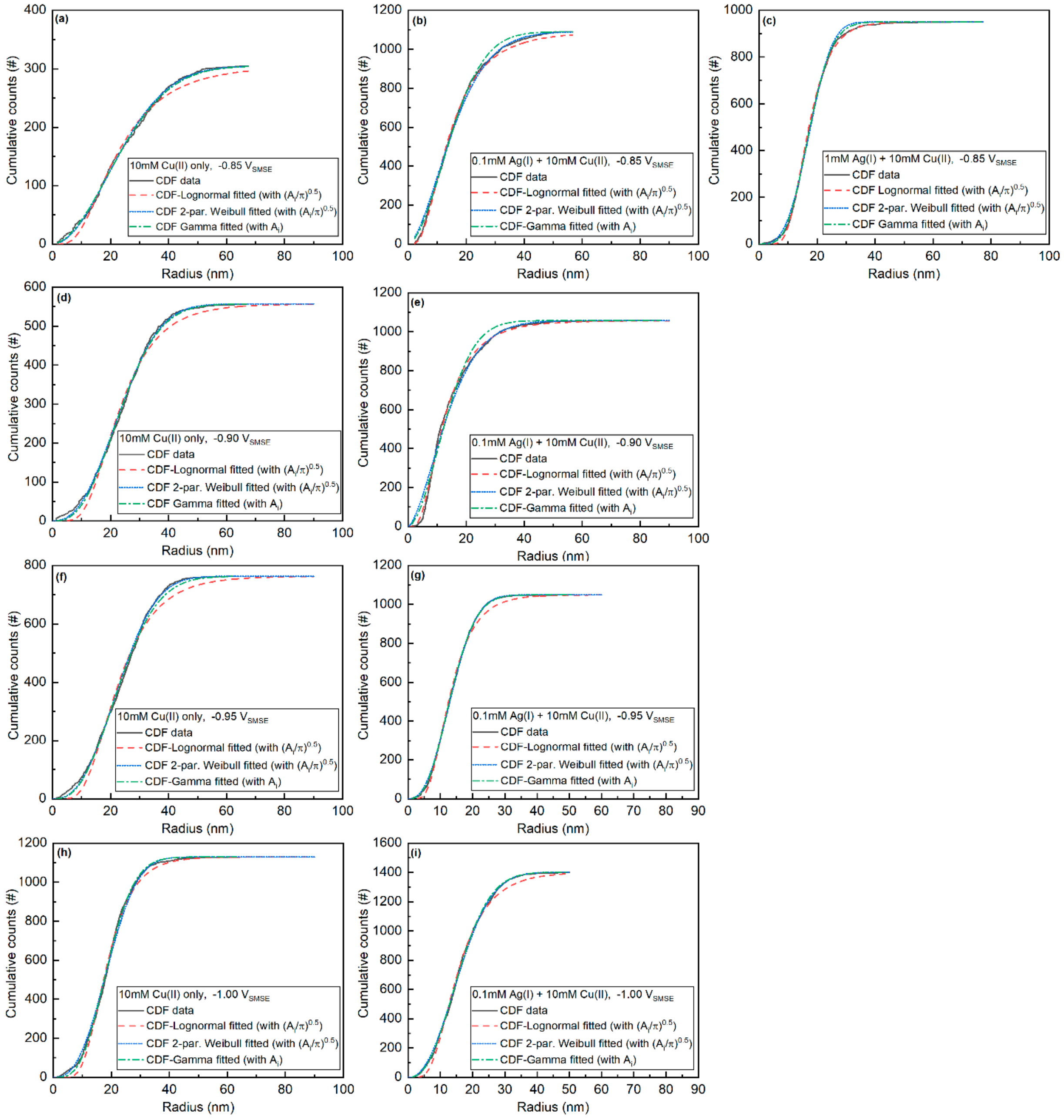


**Figure S4** CDF profile of the grain size distributions for the SEM images in **Fig.4**.

## Section S4: Statistical characteristics of the observed features

| Particle size (in radius) | Mean (nm) | Standard deviation (nm) | Skewness | Kurtosis |
|---|---|---|---|---|
| -0.85V<br>10mM Cu(II) | 24.39 | 13.01 | 0.49 | -0.13 |
| -0.90V<br>10mM Cu(II) | 23.88 | 10.81 | 0.43 | 0.67 |
| -0.95V<br>10mM Cu(II) | 23.23 | 10.05 | 0.20 | -0.06 |
| -1.00V<br>10mM Cu(II) | 19.24 | 8.01 | 0.79 | 2.13 |
| -0.85V<br>0.1mM Ag(I) + 10mM Cu(II) | 16.42 | 9.93 | 1.12 | 1.10 |
| -0.90V<br>0.1mM Ag(I) + 10mM Cu(II) | 14.75 | 9.45 | 1.77 | 5.29 |
| -0.95V<br>0.1mM Ag(I) + 10mM Cu(II) | 13.76 | 5.98 | 0.71 | 1.42 |
| -1.00V<br>0.1mM Ag(I) + 10mM Cu(II) | 16.45 | 7.74 | 0.71 | 0.75 |
| -0.85V<br>1.0mM Ag(I) + 10mM Cu(II) | 17.94 | 7.15 | 1.48 | 7.03 |

| Voronoi cell size (in radius) | Mean (nm) | Standard deviation (nm) | Skewness | Kurtosis |
|---|---|---|---|---|
| -0.85V<br>10mM Cu(II) | 76.46 | 21.06 | 0.12 | -0.13 |
| -0.90V<br>10mM Cu(II) | 56.40 | 16.31 | 0.06 | 0.19 |
| -0.95V<br>10mM Cu(II) | 48.18 | 13.22 | 0.13 | 0.01 |
| -1.00V<br>10mM Cu(II) | 39.62 | 9.79 | 0.53 | 0.83 |
| -0.85V<br>0.1mM Ag(I) + 10mM Cu(II) | 39.82 | 12.44 | 0.51 | 0.65 |
| -0.90V<br>0.1mM Ag(I) + 10mM Cu(II) | 40.53 | 12.17 | 1.21 | 3.09 |
| -0.95V<br>0.1mM Ag(I) + 10mM Cu(II) | 41.02 | 9.67 | 0.49 | 0.74 |
| -1.00V<br>0.1mM Ag(I) + 10mM Cu(II) | 35.30 | 9.74 | 0.55 | 0.66 |
| -0.85V<br>1.0mM Ag(I) + 10mM Cu(II) | 43.35 | 10.47 | 1.00 | 3.91 |

| 1st NN distance | Mean (nm) | Standard deviation (nm) | Skewness | Kurtosis |
|---|---|---|---|---|
| -0.85V<br>10mM Cu(II) | 87.01 | 28.43 | 0.50 | 0.96 |
| -0.90V<br>10mM Cu(II) | 68.24 | 19.89 | 0.42 | 0.17 |
| -0.95V<br>10mM Cu(II) | 60.73 | 15.38 | 0.57 | 0.12 |
| -1.00V<br>10mM Cu(II) | 51.92 | 11.74 | 0.16 | 0.16 |
| -0.85V<br>0.1mM Ag(I) + 10mM Cu(II) | 50.05 | 15.87 | 0.45 | 0.20 |
| -0.90V<br>0.1mM Ag(I) + 10mM Cu(II) | 50.85 | 14.64 | 0.60 | 0.44 |
| -0.95V<br>0.1mM Ag(I) + 10mM Cu(II) | 51.92 | 14.58 | 0.40 | -0.09 |
| -1.00V<br>0.1mM Ag(I) + 10mM Cu(II) | 45.87 | 12.90 | 0.48 | 0.39 |
| -0.85V<br>1.0mM Ag(I) + 10mM Cu(II) | 58.13 | 14.43 | 0.75 | 1.23 |

| 2nd NN distance | Mean (nm) | Standard deviation (nm) | Skewness | Kurtosis |
|---|---|---|---|---|
| -0.85V<br>10mM Cu(II) | 114.98 | 31.97 | 0.74 | 0.66 |
| -0.90V<br>10mM Cu(II) | 88.58 | 21.79 | 0.46 | 0.03 |
| -0.95V<br>10mM Cu(II) | 77.99 | 17.13 | 0.33 | 0.15 |
| -1.00V<br>10mM Cu(II) | 64.89 | 12.38 | 0.26 | -0.11 |
| -0.85V<br>0.1mM Ag(I) + 10mM Cu(II) | 66.01 | 15.78 | 0.28 | -0.28 |
| -0.90V<br>0.1mM Ag(I) + 10mM Cu(II) | 65.90 | 15.96 | 0.51 | 0.16 |
| -0.95V<br>0.1mM Ag(I) + 10mM Cu(II) | 67.21 | 14.36 | 0.28 | -0.30 |
| -1.00V<br>0.1mM Ag(I) + 10mM Cu(II) | 59.33 | 13.01 | 0.32 | 0.19 |
| -0.85V<br>1.0mM Ag(I) + 10mM Cu(II) | 71.96 | 14.88 | 0.49 | 0.83 |

| 5th NN distance | Mean (nm) | Standard deviation (nm) | Skewness | Kurtosis |
|---|---|---|---|---|
| -0.85V<br>10mM Cu(II) | 180.03 | 31.88 | 0.11 | 0.21 |
| -0.90V<br>10mM Cu(II) | 137.45 | 23.46 | 0.28 | -0.31 |
| -0.95V<br>10mM Cu(II) | 118.93 | 19.40 | 0.12 | -0.46 |
| -1.00V<br>10mM Cu(II) | 97.52 | 13.57 | 0.11 | -0.18 |
| -0.85V<br>0.1mM Ag(I) + 10mM Cu(II) | 101.14 | 14.78 | 0.11 | -0.14 |
| -0.90V<br>0.1mM Ag(I) + 10mM Cu(II) | 100.85 | 17.02 | 0.13 | -0.12 |
| -0.95V<br>0.1mM Ag(I) + 10mM Cu(II) | 99.72 | 14.12 | 0.27 | 0.16 |
| -1.00V<br>0.1mM Ag(I) + 10mM Cu(II) | 88.71 | 12.19 | 0.11 | 0.21 |
| -0.85V<br>1.0mM Ag(I) + 10mM Cu(II) | 105.15 | 15.43 | 0.43 | 0.98 |

| 10th NN distance | Mean (nm) | Standard deviation (nm) | Skewness | Kurtosis |
|---|---|---|---|---|
| -0.85V<br>10mM Cu(II) | 256.05 | 29.50 | 0.55 | 0.79 |
| -0.90V<br>10mM Cu(II) | 191.04 | 23.26 | 0.14 | -0.48 |
| -0.95V<br>10mM Cu(II) | 165.90 | 19.02 | -0.12 | 0.50 |
| -1.00V<br>10mM Cu(II) | 137.27 | 12.41 | 0.09 | 0.36 |
| -0.85V<br>0.1mM Ag(I) + 10mM Cu(II) | 140.43 | 14.29 | 0.07 | -0.31 |
| -0.90V<br>0.1mM Ag(I) + 10mM Cu(II) | 140.38 | 17.06 | 0.24 | -0.22 |
| -0.95V<br>0.1mM Ag(I) + 10mM Cu(II) | 139.50 | 14.20 | 0.39 | -0.24 |
| -1.00V<br>0.1mM Ag(I) + 10mM Cu(II) | 123.68 | 12.66 | -0.02 | -0.14 |
| -0.85V<br>1.0mM Ag(I) + 10mM Cu(II) | 147.09 | 16.46 | 0.34 | 0.13 |

| Coordination number | Mean (#) | Standard deviation (#) | Skewness | Kurtosis |
|---|---|---|---|---|
| -0.85V<br>10mM Cu(II) | 6.004 | 7.287 | 0.079 | -1.068 |
| -0.90V<br>10mM Cu(II) | 5.919 | 10.638 | -0.007 | -1.184 |
| -0.95V<br>10mM Cu(II) | 5.953 | 11.589 | 0.064 | -1.133 |
| -1.00V<br>10mM Cu(II) | 5.956 | 12.832 | 0.141 | -0.793 |
| -0.85V<br>0.1mM Ag(I) + 10mM Cu(II) | 5.989 | 14.326 | 0.141 | -1.160 |
| -0.90V<br>0.1mM Ag(I) + 10mM Cu(II) | 5.945 | 13.323 | 0.116 | -1.029 |
| -0.95V<br>0.1mM Ag(I) + 10mM Cu(II) | 5.951 | 11.965 | 0.065 | -0.958 |
| -1.00V<br>0.1mM Ag(I) + 10mM Cu(II) | 5.952 | 15.083 | 0.065 | -1.020 |
| -0.85V<br>1.0mM Ag(I) + 10mM Cu(II) | 5.984 | 10.611 | 0.115 | -0.616 |

| Voronoi cell occupancy | Mean ($\%_{area}$) | Standard deviation ($\%_{area}$) | Skewness | Kurtosis |
|---|---|---|---|---|
| -0.85V<br>10mM Cu(II) | 11.81 | 9.15 | 1.07 | 0.91 |
| -0.90V<br>10mM Cu(II) | 19.14 | 11.20 | 0.58 | 0.14 |
| -0.95V<br>10mM Cu(II) | 24.75 | 13.45 | 0.40 | -0.05 |
| -1.00V<br>10mM Cu(II) | 24.37 | 11.82 | 0.35 | 0.01 |
| -0.85V<br>0.1mM Ag(I) + 10mM Cu(II) | 16.55 | 11.66 | 0.92 | 0.89 |
| -0.90V<br>0.1mM Ag(I) + 10mM Cu(II) | 13.32 | 9.61 | 1.17 | 1.45 |
| -0.95V<br>0.1mM Ag(I) + 10mM Cu(II) | 11.53 | 6.29 | 0.79 | 0.99 |
| -1.00V<br>0.1mM Ag(I) + 10mM Cu(II) | 21.92 | 11.47 | 0.37 | -0.37 |
| -0.85V<br>1.0mM Ag(I) + 10mM Cu(II) | 17.13 | 6.75 | 0.31 | 0.12 |

## Section S5 Summary of fitting results

The cumulative distribution function (CDF) of the Lognormal distribution for feature $R$, with two fitting parameters ($\gamma_L$, $k_L$):

$$F(R) = \frac{N_{tot}}{2}\left(1 + \operatorname{erf}\left(\frac{k_L \ln(R/\gamma_L R_0)}{\sqrt{2}}\right)\right)$$

The CDF of the Weibull distribution for feature $R$, with two fitting parameters ($\gamma_W$, $k_W$):

$$F(R) = N_{tot}\left(1 - \exp\left(-\left(\frac{R}{\gamma_W R_0}\right)^{k_W}\right)\right)$$

The CDF of the Gamma distribution for feature $A_R$, with two fitting parameters ($\alpha'$, $\gamma_G$):

$$F(A_R) = \frac{N^{tot}}{\Gamma(\alpha')}\left(\frac{\gamma_G}{A_0}\right)^{\alpha'} \int_{o}^{A_R} A^{\alpha'-1} \exp\left(-\frac{\gamma_G A}{A_0}\right) dA$$

Note that $R_0$ and $A_0$ are the average value of the feature $R$. Using average radius R as an example, $R_0$ is defined as:

$$R_0 = \frac{\sum_i R_i}{N^{tot}}$$

Where $N^{tot}$ is total number of particles identified in the SEM image.

# 1. Particle size

**Table S1a** Fitting results of the particle size distribution of the deposits in **Fig.9**

| Particle size | Weibull ($R$) | | Lognormal ($R$) | | Gamma ($R^2$) | |
|---|---|---|---|---|---|---|
| | $\gamma_W$ | $k_W$ | $\gamma_L$ | $k_L$ | $\gamma_G$ | $\alpha'$ |
| 10mM Cu(II), -0.85V | 1.12987 | 1.97089 | 0.90767 | 1.69584 | 0.90760 | 0.92953 |
| 10mM Cu(II), -0.90V | 1.12542 | 2.43890 | 0.95418 | 2.18174 | 1.42000 | 1.43376 |
| 10mM Cu(II), -0.95V | 1.12792 | 2.54006 | 0.95873 | 2.15880 | 1.35661 | 1.41381 |
| 10mM Cu(II), -1.00V | 1.11847 | 2.65641 | 0.95424 | 2.54298 | 1.94835 | 1.87777 |
| 10mM Cu(II) + 0.1mM Ag(I), -0.85V | 1.10658 | 1.63926 | 0.85323 | 1.55664 | 0.96770 | 0.86407 |
| 10mM Cu(II) + 0.1mM Ag(I), -0.90V | 1.08407 | 1.55333 | 0.82237 | 1.60110 | 1.09406 | 0.87531 |
| 10mM Cu(II) + 0.1mM Ag(I), -0.95V | 1.12282 | 2.47259 | 0.93677 | 2.17052 | 1.45187 | 1.43831 |
| 10mM Cu(II) + 0.1mM Ag(I), -1.00V | 1.12401 | 2.24908 | 0.92584 | 2.03861 | 1.31336 | 1.28070 |
| 10mM Cu(II) + 1mM Ag(I), -0.85V | 1.08417 | 3.16925 | 0.93590 | 2.75953 | 2.34142 | 2.18333 |

**Table S1b** Goodness of fitting (reflected by the value of reduced chi-square). Best-fitted results are highlighted in the table.

| Particle size ($R^2$) | Lognormal | Weibull | Gamma |
|---|---|---|---|
| 10mM Cu(II), -0.85V | 76.82417 | **17.54486** | **17.23666** |
| 10mM Cu(II), -0.90V | 314.40398 | **33.70238** | **59.57201** |
| 10mM Cu(II), -0.95V | 678.58269 | **85.73903** | **145.33532** |
| 10mM Cu(II), -1.00V | 704.44851 | **175.32914** | **184.43372** |
| 10mM Cu(II) + 0.1mM Ag(I), -0.85V | **214.48689** | **179.32499** | 337.10703 |
| 10mM Cu(II) + 0.1mM Ag(I), -0.90V | **439.99152** | 882.90216 | 1968.32421 |
| 10mM Cu(II) + 0.1mM Ag(I), -0.95V | 400.91358 | **39.46859** | **35.19554** |
| 10mM Cu(II) + 0.1mM Ag(I), -1.00V | 785.37822 | **187.71873** | **194.52263** |
| 10mM Cu(II) + 1mM Ag(I), -0.85V | **82.86434** | 205.1378 | **61.38359** |

## 2. Voronoi cell size

**Table S2a** Voronoi cell size fitted parameters

| Voronoi cell size | Weibull ($R$) | | Lognormal ($R$) | | Gamma ($R^2$) | |
|---|---|---|---|---|---|---|
| | $\gamma_W$ | $k_W$ | $\gamma_L$ | $k_L$ | $\gamma_G$ | $\alpha'$ |
| 10mM Cu(II), -0.85V | 1.05289 | 4.09502 | 0.94885 | 3.61167 | 3.44035 | 3.47088 |
| 10mM Cu(II), -0.90V | 1.05154 | 4.05645 | 0.93161 | 3.62056 | 3.30820 | 3.33436 |
| 10mM Cu(II), -0.95V | 1.05133 | 4.21264 | 0.93442 | 3.76194 | 3.61725 | 3.63806 |
| 10mM Cu(II), -1.00V | 1.04224 | 4.75414 | 0.94301 | 4.15507 | 4.56086 | 4.51131 |
| 10mM Cu(II) + 0.1mM Ag(I), -0.85V | 1.03378 | 3.80001 | 0.91514 | 3.32303 | 3.08137 | 3.00337 |
| 10mM Cu(II) + 0.1mM Ag(I), -0.90V | 1.01579 | 4.03190 | 0.91605 | 3.44566 | 3.62628 | 3.39955 |
| 10mM Cu(II) + 0.1mM Ag(I), -0.95V | 1.03866 | 4.91986 | 0.94825 | 4.34650 | 5.00855 | 4.91622 |
| 10mM Cu(II) + 0.1mM Ag(I), -1.00V | 1.03940 | 4.14152 | 0.93085 | 3.73009 | 3.60812 | 3.54397 |
| 10mM Cu(II) + 1mM Ag(I), -0.85V | 1.03547 | 5.00922 | 0.94511 | 4.17150 | 5.14593 | 5.02626 |

**Table S2b** Goodness of fitting (reflected by the value of reduced chi-square). Best fitted results are highlighted in the table.

| Voronoi cell size ($R^2$) | Lognormal | Weibull | Gamma |
|---|---|---|---|
| 10mM Cu(II), -0.85V | **12.5026** | **13.48914** | 20.97627 |
| 10mM Cu(II), -0.90V | **4.06918** | 22.23003 | 78.47933 |
| 10mM Cu(II), -0.95V | **3.07649** | 41.92228 | 81.85295 |
| 10mM Cu(II), -1.00V | **0.50762** | 153.71785 | 91.80609 |
| 10mM Cu(II) + 0.1mM Ag(I), -0.85V | **1.21227** | 300.57755 | 68.38255 |
| 10mM Cu(II) + 0.1mM Ag(I), -0.90V | **0.26581** | 979.38649 | 244.08844 |
| 10mM Cu(II) + 0.1mM Ag(I), -0.95V | **0.20933** | 463.80098 | 31.60001 |
| 10mM Cu(II) + 0.1mM Ag(I), -1.00V | **0.39089** | 602.76994 | 79.01999 |
| 10mM Cu(II) + 1mM Ag(I), -0.85V | **0.25940** | 248.90517 | 16.12252 |

## 3. 1st nearest neighbor distance

**Table S3a** 1st nearest neighbor distance fitted parameters

| 1st nearest neighbor distance | Weibull ($R$) | | Lognormal ($R$) | | Gamma ($R^2$) | |
|---|---|---|---|---|---|---|
| | $\gamma_W$ | $k_W$ | $\gamma_L$ | $k_L$ | $\gamma_G$ | $\alpha'$ |
| 10mM Cu(II), -0.85V | 1.07442 | 3.79652 | 0.96007 | 3.31323 | 3.19153 | 3.03407 |
| 10mM Cu(II), -0.90V | 1.08401 | 3.89880 | 0.96985 | 3.36270 | 3.17277 | 3.13083 |
| 10mM Cu(II), -0.95V | 1.07092 | 4.30908 | 0.96850 | 3.82405 | 4.00653 | 3.91415 |
| 10mM Cu(II), -1.00V | 1.07188 | 5.07042 | 0.98463 | 4.46269 | 5.25076 | 5.22543 |
| 10mM Cu(II) + 0.1mM Ag(I), -0.85V | 1.09141 | 3.55359 | 0.96632 | 3.08794 | 2.69432 | 2.66291 |
| 10mM Cu(II) + 0.1mM Ag(I), -0.90V | 1.08170 | 3.89419 | 0.96704 | 3.37268 | 3.19299 | 3.13916 |
| 10mM Cu(II) + 0.1mM Ag(I), -0.95V | 1.08255 | 3.93216 | 0.96950 | 3.45077 | 3.28200 | 3.24035 |
| 10mM Cu(II) + 0.1mM Ag(I), -1.00V | 1.08349 | 4.01786 | 0.97187 | 3.47295 | 3.34836 | 3.31379 |
| 10mM Cu(II) + 1mM Ag(I), -0.85V | 1.06616 | 4.57076 | 0.96897 | 4.03372 | 4.46957 | 4.33880 |

**Table S3b** Goodness of fitting (reflected by the value of reduced chi-square). Best fitted results are highlighted in the table.

| 1st nearest neighbor distance ($R^2$) | Lognormal | Weibull | Gamma |
|---|---|---|---|
| 10mM Cu(II), -0.85V | **6.95421** | 30.83670 | **13.04475** |
| 10mM Cu(II), -0.90V | 46.43307 | 50.47370 | **17.13337** |
| 10mM Cu(II), -0.95V | **56.33470** | 362.67955 | **145.20994** |
| 10mM Cu(II), -1.00V | **76.50000** | 383.51553 | **69.34010** |
| 10mM Cu(II) + 0.1mM Ag(I), -0.85V | 182.92772 | 161.42956 | **40.17723** |
| 10mM Cu(II) + 0.1mM Ag(I), -0.90V | 123.35001 | 239.17917 | **73.73904** |
| 10mM Cu(II) + 0.1mM Ag(I), -0.95V | **72.13299** | 378.45089 | **122.48618** |
| 10mM Cu(II) + 0.1mM Ag(I), -1.00V | 297.70254 | 330.83611 | **105.23790** |
| 10mM Cu(II) + 1mM Ag(I), -0.85V | **63.78937** | 569.24604 | 213.37346 |

## 4. 2nd nearest neighbor distance

**Table S4a** 2nd nearest neighbor distance fitted parameters

| 2nd nearest neighbor distance | Weibull ($R$) | | Lognormal ($R$) | | Gamma ($R^2$) | |
|---|---|---|---|---|---|---|
| | $\gamma_W$ | $k_W$ | $\gamma_L$ | $k_L$ | $\gamma_G$ | $\alpha'$ |
| 10mM Cu(II), -0.85V | 1.07091 | 4.10578 | 0.96333 | 3.53895 | 3.56725 | 3.45576 |
| 10mM Cu(II), -0.90V | 1.07267 | 4.48601 | 0.99625 | 3.95179 | 4.2292 | 4.16438 |
| 10mM Cu(II), -0.95V | 1.06925 | 5.21588 | 0.98386 | 4.49580 | 5.38779 | 5.35998 |
| 10mM Cu(II), -1.00V | 1.06311 | 5.94565 | 0.98822 | 5.11797 | 6.88030 | 6.86881 |
| 10mM Cu(II) + 0.1mM Ag(I), -0.85V | 1.07748 | 4.53512 | 0.97870 | 3.99441 | 4.25542 | 4.24055 |
| 10mM Cu(II) + 0.1mM Ag(I), -0.90V | 1.06908 | 4.66717 | 0.97454 | 4.08097 | 4.52443 | 4.44175 |
| 10mM Cu(II) + 0.1mM Ag(I), -0.95V | 1.06948 | 5.09897 | 0.98215 | 4.46090 | 5.27564 | 5.25186 |
| 10mM Cu(II) + 0.1mM Ag(I), -1.00V | 1.06944 | 5.16101 | 0.98331 | 4.50187 | 5.37672 | 5.34460 |
| 10mM Cu(II) + 1mM Ag(I), -0.85V | 1.06522 | 5.45188 | 0.98364 | 4.76395 | 6.00192 | 5.95536 |

**Table S4b** Goodness of fitting (reflected by the value of reduced chi-square). Best fitted results are highlighted in the table.

| 2nd nearest neighbor distance ($R^2$) | Lognormal | Weibull | Gamma |
|---|---|---|---|
| 10mM Cu(II), -0.85V | **6.51241** | 18.83942 | **6.31931** |
| 10mM Cu(II), -0.90V | **16.01587** | 109.6247 | **32.22873** |
| 10mM Cu(II), -0.95V | 69.83068 | 83.54715 | **27.12900** |
| 10mM Cu(II), -1.00V | 157.68637 | 207.43057 | **57.21822** |
| 10mM Cu(II) + 0.1mM Ag(I), -0.85V | **83.11630** | 371.20762 | **101.73362** |
| 10mM Cu(II) + 0.1mM Ag(I), -0.90V | **42.24740** | 386.37557 | **91.34833** |
| 10mM Cu(II) + 0.1mM Ag(I), -0.95V | **52.64047** | 352.10098 | **59.70631** |
| 10mM Cu(II) + 0.1mM Ag(I), -1.00V | **120.10369** | 517.14598 | **84.19707** |
| 10mM Cu(II) + 1mM Ag(I), -0.85V | **48.88195** | 225.06924 | **29.13834** |

## 5. 5th nearest neighbor distance

**Table S5a** 5th nearest neighbor distance fitted parameters

| 5th nearest neighbor distance | Weibull ($R$) | | Lognormal ($R$) | | Gamma ($R^2$) | |
|---|---|---|---|---|---|---|
| | $\gamma_W$ | $k_W$ | $\gamma_L$ | $k_L$ | $\gamma_G$ | $\alpha'$ |
| 10mM Cu(II), -0.85V | 1.06169 | 6.51987 | 0.99348 | 5.65419 | 8.36297 | 8.36532 |
| 10mM Cu(II), -0.90V | 1.05837 | 6.30849 | 0.98678 | 5.49028 | 7.86605 | 7.83635 |
| 10mM Cu(II), -0.95V | 1.05900 | 6.63327 | 0.99174 | 5.83908 | 8.72582 | 8.74774 |
| 10mM Cu(II), -1.00V | 1.04415 | 8.59413 | 0.99452 | 7.07614 | 12.78786 | 13.21044 |
| 10mM Cu(II) + 0.1mM Ag(I), -0.85V | 1.05051 | 7.85100 | 0.99459 | 6.79256 | 11.81234 | 11.82001 |
| 10mM Cu(II) + 0.1mM Ag(I), -0.90V | 1.06002 | 6.65224 | 0.99259 | 5.73625 | 8.50450 | 8.53542 |
| 10mM Cu(II) + 0.1mM Ag(I), -0.95V | 1.04824 | 7.92292 | 0.99238 | 6.95991 | 12.39202 | 12.35253 |
| 10mM Cu(II) + 0.1mM Ag(I), -1.00V | 1.0463 | 8.56217 | 0.99524 | 7.51823 | 14.36227 | 14.34471 |
| 10mM Cu(II) + 1mM Ag(I), -0.85V | 1.04563 | 8.02095 | 0.99076 | 6.97739 | 12.58901 | 12.48448 |

**Table S5b** Goodness of fitting (reflected by the value of reduced chi-square). Best fitted results are highlighted in the table.

| 5th nearest neighbor distance ($R^2$) | Lognormal | Weibull | Gamma |
|---|---|---|---|
| 10mM Cu(II), -0.85V | 11.25316 | **4.16373** | **5.16774** |
| 10mM Cu(II), -0.90V | **31.95991** | 87.94815 | **34.50869** |
| 10mM Cu(II), -0.95V | **41.86595** | 120.98114 | **25.90731** |
| 10mM Cu(II), -1.00V | **54.51746** | 201.79084 | **35.25198** |
| 10mM Cu(II) + 0.1mM Ag(I), -0.85V | **90.51654** | 168.75814 | **34.97838** |
| 10mM Cu(II) + 0.1mM Ag(I), -0.90V | 143.7591 | 116.78033 | **63.31640** |
| 10mM Cu(II) + 0.1mM Ag(I), -0.95V | **33.14437** | 260.67084 | **35.23964** |
| 10mM Cu(II) + 0.1mM Ag(I), -1.00V | **94.16697** | 508.74290 | **72.12255** |
| 10mM Cu(II) + 1mM Ag(I), -0.85V | **22.55905** | 259.41360 | **25.99566** |

# 6. 10th nearest neighbor distance

**Table S6a** 10th nearest neighbor distance fitted parameters

| 10th nearest neighbor distance | Weibull ($R$) | | Lognormal ($R$) | | Gamma ($R^2$) | |
|---|---|---|---|---|---|---|
| | $\gamma_W$ | $k_W$ | $\gamma_L$ | $k_L$ | $\gamma_G$ | $\alpha'$ |
| 10mM Cu(II), -0.85V | 1.03068 | 10.45879 | 0.98944 | 9.34230 | 22.34817 | 21.97758 |
| 10mM Cu(II), -0.90V | 1.04347 | 8.80726 | 0.99322 | 7.84437 | 15.60722 | 15.56108 |
| 10mM Cu(II), -0.95V | 1.04063 | 10.63479 | 0.99934 | 9.18780 | 21.31828 | 21.40040 |
| 10mM Cu(II), -1.00V | 1.03116 | 13.31070 | 0.99838 | 11.58346 | 33.76866 | 33.77081 |
| 10mM Cu(II) + 0.1mM Ag(I), -0.85V | 1.03735 | 11.0321 | 0.99733 | 9.52835 | 22.99701 | 23.02839 |
| 10mM Cu(II) + 0.1mM Ag(I), -0.90V | 1.04159 | 9.21163 | 0.99365 | 8.02439 | 16.44656 | 16.39098 |
| 10mM Cu(II) + 0.1mM Ag(I), -0.95V | 1.03097 | 11.04403 | 0.99155 | 9.72763 | 24.19991 | 23.93456 |
| 10mM Cu(II) + 0.1mM Ag(I), -1.00V | 1.03809 | 11.06642 | 0.99823 | 9.59049 | 23.23152 | 23.29686 |
| 10mM Cu(II) + 1mM Ag(I), -0.85V | 1.03884 | 9.90710 | 0.99396 | 8.58148 | 18.82291 | 18.75860 |

**Table S6b** Goodness of fitting (reflected by the value of reduced chi-square). Best fitted results are highlighted in the table.

| 10th nearest neighbor distance ($R^2$) | Lognormal | Weibull | Gamma |
|---|---|---|---|
| 10mM Cu(II), -0.85V | **9.29948** | 30.50684 | **11.79879** |
| 10mM Cu(II), -0.90V | **17.96297** | 85.68206 | **23.27756** |
| 10mM Cu(II), -0.95V | 48.33878 | 41.26070 | **25.96019** |
| 10mM Cu(II), -1.00V | **60.78702** | 176.62761 | **42.71459** |
| 10mM Cu(II) + 0.1mM Ag(I), -0.85V | **65.63527** | 174.73055 | **35.95656** |
| 10mM Cu(II) + 0.1mM Ag(I), -0.90V | **13.53730** | 210.42230 | **11.95216** |
| 10mM Cu(II) + 0.1mM Ag(I), -0.95V | **76.29233** | 553.88154 | **23.93456** |
| 10mM Cu(II) + 0.1mM Ag(I), -1.00V | **108.68298** | 267.53559 | **46.63068** |
| 10mM Cu(II) + 1mM Ag(I), -0.85V | **37.51865** | 193.01249 | **33.57950** |

# 7. Coordination number

**Table S7a** Coordination number fitted parameters

| Coordination number | Weibull (#) | | Lognormal (#) | |
|---|---|---|---|---|
| | $\gamma_W$ | $k_W$ | $\gamma_L$ | $k_L$ |
| 10mM Cu(II), -0.85V | 1.05243 | 4.52299 | 0.98847 | 3.73148 |
| 10mM Cu(II), -0.90V | 1.07828 | 4.24416 | 1.00623 | 3.32568 |
| 10mM Cu(II), -0.95V | 1.05863 | 4.50610 | 0.99549 | 3.90793 |
| 10mM Cu(II), -1.00V | 1.04041 | 4.86615 | 0.98445 | 4.33032 |
| 10mM Cu(II) + 0.1mM Ag(I), -0.85V | 1.04150 | 4.41768 | 0.98169 | 3.89484 |
| 10mM Cu(II) + 0.1mM Ag(I), -0.90V | 1.04143 | 4.45299 | 0.97718 | 3.90686 |
| 10mM Cu(II) + 0.1mM Ag(I), -0.95V | 1.04136 | 5.30097 | 0.99009 | 4.65095 |
| 10mM Cu(II) + 0.1mM Ag(I), -1.00V | 1.04731 | 4.62329 | 0.98578 | 3.96212 |
| 10mM Cu(II) + 1mM Ag(I), -0.85V | 1.03349 | 5.41587 | 0.98603 | 4.82462 |

**Table S7b** Goodness of fitting (reflected by the value of reduced chi-square). Best fitted results are highlighted in the table.

| Coordination number (#) | Lognormal | Weibull |
|---|---|---|
| 10mM Cu(II), -0.85V | **2.00561E-4** | 3.74048E-4 |
| 10mM Cu(II), -0.90V | 5.89064E-4 | **2.81596E-4** |
| 10mM Cu(II), -0.95V | **8.89230E-5** | 1.96721E-4 |
| 10mM Cu(II), -1.00V | **2.38815E-5** | 3.74904E-4 |
| 10mM Cu(II) + 0.1mM Ag(I), -0.85V | **4.97329E-5** | 3.00103E-4 |
| 10mM Cu(II) + 0.1mM Ag(I), -0.90V | **2.61589E-5** | 4.38461E-4 |
| 10mM Cu(II) + 0.1mM Ag(I), -0.95V | **7.72067E-5** | 2.87891E-4 |
| 10mM Cu(II) + 0.1mM Ag(I), -1.00V | **8.13826E-5** | 2.43556E-4 |
| 10mM Cu(II) + 1mM Ag(I), -0.85V | **7.37872E-5** | 3.64700E-4 |

# 8. Voronoi cell occupancy

**Table S8a** Voronoi cell occupancy fitted parameters

| Voronoi cell occupancy | Weibull ($R$) | | Lognormal ($R$) | | Gamma ($R^2$) | |
|---|---|---|---|---|---|---|
| | $\gamma_W$ | $k_W$ | $\gamma_L$ | $k_L$ | $\gamma_G$ | $\alpha'$ |
| 10mM Cu(II), -0.85V | 1.09200 | 1.25809 | 0.77794 | 1.10347 | 1.38815 | 1.43862 |
| 10mM Cu(II), -0.90V | 1.14749 | 1.7438 | 0.89458 | 1.49191 | 2.35974 | 2.48281 |
| 10mM Cu(II), -0.95V | 1.15610 | 1.88019 | 0.91857 | 1.62022 | 2.71266 | 2.86440 |
| 10mM Cu(II), -1.00V | 1.14272 | 2.23379 | 0.94268 | 1.94420 | 3.83393 | 3.99241 |
| 10mM Cu(II) + 0.1mM Ag(I), -0.85V | 1.12282 | 1.36463 | 0.81687 | 1.18282 | 1.56321 | 1.64387 |
| 10mM Cu(II) + 0.1mM Ag(I), -0.90V | 1.08159 | 1.36545 | 0.78319 | 1.20198 | 1.68412 | 1.69500 |
| 10mM Cu(II) + 0.1mM Ag(I), -0.95V | 1.12544 | 1.92729 | 0.89914 | 1.68549 | 3.00344 | 3.07514 |
| 10mM Cu(II) + 0.1mM Ag(I), -1.00V | 1.14935 | 1.90802 | 0.91346 | 1.64796 | 2.83528 | 2.96879 |
| 10mM Cu(II) + 1mM Ag(I), -0.85V | 1.11416 | 2.85079 | 0.95775 | 2.48311 | 2.67300 | 2.72143 |

**Table S8b** Goodness of fitting (reflected by the value of reduced chi-square). Best fitted results are highlighted in the table.

| Voronoi cell occupancy ($\%_{area}$) | Lognormal | Weibull | Gamma |
|---|---|---|---|
| 10mM Cu(II), -0.85V | 83.01969 | **17.52204** | **23.56213** |
| 10mM Cu(II), -0.90V | 443.36252 | **96.96739** | 197.33850 |
| 10mM Cu(II), -0.95V | 822.81991 | **145.32177** | 363.57820 |
| 10mM Cu(II), -1.00V | 1153.98342 | **111.66994** | 502.23432 |
| 10mM Cu(II) + 0.1mM Ag(I), -0.85V | 1225.90952 | **113.03177** | 261.30803 |
| 10mM Cu(II) + 0.1mM Ag(I), -0.90V | **240.22976** | 428.13348 | **278.25982** |
| 10mM Cu(II) + 0.1mM Ag(I), -0.95V | 510.59526 | **27.99956** | 113.48462 |
| 10mM Cu(II) + 0.1mM Ag(I), -1.00V | 1990.49278 | **222.02641** | 758.58013 |
| 10mM Cu(II) + 1mM Ag(I), -0.85V | 286.26469 | **44.38022** | **84.55129** |

# Section S6 Mean and standard deviation of the Voronoi cell occupancy and the coordination number

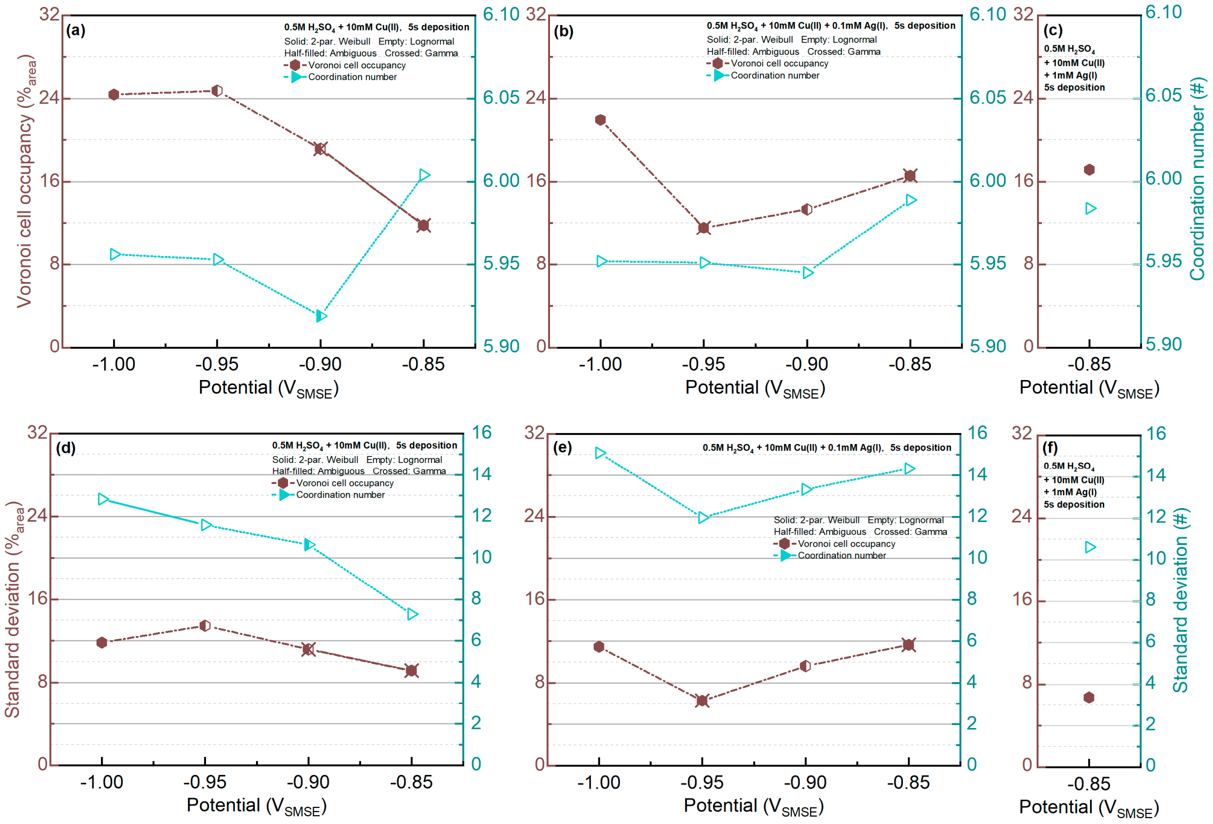


**Figure S5,** Mean and standard deviation of Voronoi cell occupancy and coordination number.

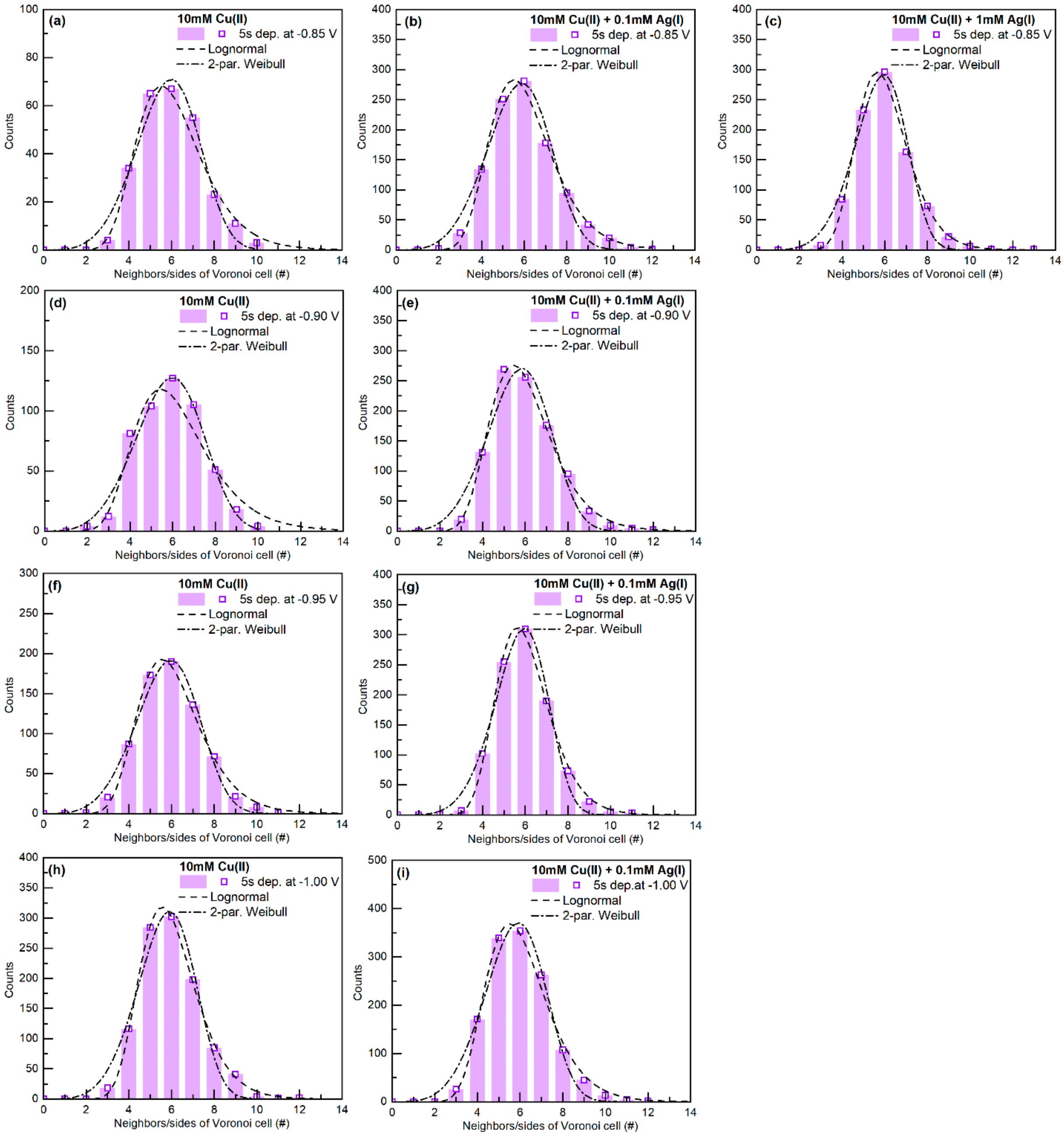


**Figure S6** Voronoi cell coordination number. Supporting electrolyte is 0.5M $H_2SO_4$. The fitting was conducted directly with PDF. Reference electrode of SMSE was used for the potentials.
